# Single Nucleotide Polymorphisms from *CSF2*, *FLT1*, *TFPI* and *TLR9* Genes Are Associated with Prelabor Rupture of Membranes

**DOI:** 10.3390/genes12111725

**Published:** 2021-10-28

**Authors:** Wioletta Izabela Wujcicka, Marian Kacerovsky, Michał Krekora, Piotr Kaczmarek, Mariusz Grzesiak

**Affiliations:** 1 Scientific Laboratory of the Center of Medical Laboratory Diagnostics and Screening, Polish Mother’s Memorial Hospital-Research Institute, 281/289 Rzgowska St., 93-338 Lodz, Poland; 2 Department of Obstetrics and Gynecology, University Hospital Hradec Kralove, Faculty of Medicine in Hradec Kralove, Charles University, Simkova 870, 500 03 Hradec Kralove, Czech Republic; marian.kacerovsky@gmail.com; 3 Biomedical Research Center, University Hospital Hradec Kralove, 500 03 Hradec Kralove, Czech Republic; 4 Department of Obstetrics and Gynecology, Polish Mother’s Memorial Hospital-Research Institute, 93-338 Lodz, Poland; krekoram@poczta.onet.pl; 5 Department of Gynecology and Obstetrics, Medical University of Lodz, 281/289 Rzgowska St., 93-338 Lodz, Poland; mariusz.grzesiak@gmail.com; 6 Laboratory of Prenatal Fetal and Maternal Diagnostics, Polish Mother’s Memorial Hospital-Research Institute, 93-338 Lodz, Poland; kaczmarekpiotr1@gmail.com; 7 Department of Perinatology, Obstetrics and Gynecology, Polish Mother’s Memorial Hospital-Research Institute, 93-338 Lodz, Poland

**Keywords:** prelabor rupture of membranes (PROM), pPROM, tPROM, hemostasis, angiogenesis, pregnancy, genotyping, single nucleotide polymorphism (SNP), restriction fragment length polymorphism (RFLP)

## Abstract

A prelabor rupture of membranes (PROM) and its subtypes, preterm PROM (pPROM) and term PROM (tPROM), are associated with disturbances in the hemostatic system and angiogenesis. This study was designed to demonstrate the role of single nucleotide polymorphisms (SNPs), localized in *CSF2* (rs25881), *FLT1* (rs722503), *TFPI* (C-399T) and *TLR9* (rs352140) genes, in PROM. A population of 360 women with singleton pregnancy consisted of 180 PROM cases and 180 healthy controls. A single-SNP analysis showed a similar distribution of genotypes in the studied polymorphisms between the PROM or the pPROM women and the healthy controls. Double-SNP TT variants for *CSF2* and *FLT1* polymorphisms, CC variants for *TLR9* and *TFPI* SNPs, TTC for *CSF2*, *FLT1* and *TLR9* polymorphisms, TTT for *FLT1*, *TLR9* and *TFPI* SNPs and CCCC and TTTC complex variants for all tested SNPs correlated with an increased risk of PROM after adjusting for APTT, PLT parameters and/or pregnancy disorders. The TCT variants for the *CSF2*, *FLT1* and *TLR9* SNPs and the CCTC for the *CSF2*, *FLT1*, *TLR9* and *TFPI* polymorphisms correlated with a reduced risk of PROM when corrected by PLT and APTT, respectively. We concluded that the polymorphisms of genes, involved in hemostasis and angiogenesis, contributed to PROM.

## 1. Introduction

A prelabor rupture of membranes (PROM) concerns the membranes with amniotic fluid leakage that occurs before the onset of labor or regular uterine activity and is observed in approximately 40% of preterm deliveries [1,2,3]. Based on the gestational age at the time of membrane rupture, the PROM is divided into a preterm PROM (pPROM), if the disturbance occurs before the 37th week, and the term PROM (tPROM), observed from the 37th week [1]. TPROM is diagnosed in roughly 8% of full-term pregnancies, followed by adverse maternal and perinatal outcomes, including placental abruption, cord compression, cord prolapse, a risk of caesarean delivery and maternal and neonatal infection [1,2,4]. In turn, pPROM affects about 3–4% of all births, causing one-third of all preterm labors and being one of the most serious complications of pregnancy [5,6,7]. The occurrence of pPROM is accompanied by increased maternal and offspring morbidity, as well as fetal and neonatal mortality [8,9,10]. PPROM is associated with an increased risk of intra-amniotic infection, acute and chronic histological chorioamnionitis, clinical chorioamnionitis, cord prolapse, placental abruption and postpartum endometritis [8,9,10,11]. The complications in neonates include pulmonary hypoplasia, respiratory distress syndrome (RDS), bronchopulmonary dysplasia (BPD), adverse neurodevelopmental outcomes, intraventricular hemorrhage (IVH), retinopathy of prematurity (ROP), cardiovascular diseases, necrotizing enterocolitis (NEC), sepsis and death [9,10].

Disorders in the hemostatic system and angiogenesis have been reported among the factors, which determine the occurrence of PROM [12,13,14,15]. In vitro studies have shown thrombin participation in fetal-membrane-weakening processes, accompanied by their remodeling and apoptosis, similar to the changes that occur physiologically fetal membranes in term pregnancies [16,17]. Thrombin can compromise the membranes, both directly, affecting the extracellular matrix by converting matrix metalloproteinase (MMP) proteins from their precursor forms to the active form of the enzyme, and indirectly, via protease-activated receptors (PAR1, 3 and 4) [18,19,20,21,22]. In the case of tissue factor (TF), which initiates the activation of blood clotting through thrombin synthesis, it has been found that an increased expression is associated with pPROM [13,23]. In turn, the tissue factor pathway inhibitor (TFPI) that prevents the TF-dependent coagulation pathway has been found to be reduced in women with pPROM, compared to full-term pregnancies [13]. The granulocyte-macrophage colony-stimulating factor (GM-CSF, CSF2) has also been reported to be involved in fetal membrane weakening and is characterized by an increased activity in the fetal membranes with PROM, compared to labor on time [15,24].

The most common infections associated with pPROM are caused by genital mycoplasmas: Ureaplasma and Mycoplasma hominis [25,26,27,28]. The toll-like receptors (TLRs) are the key molecules in the immune response against a variety of microorganisms, including bacteria [29,30,31]. Increased levels of amniotic-fluid-soluble forms of TLR2 (sTLR2) and TLR4 (sTLR4) have been found in women with pPROM in the case of microbial invasion of the amniotic cavity (MIAC), determined by the presence of PCR products for the genital mycoplasmas (Ureaplasma spp. and M. hominis), Chlamydia trachomatis and/or the growth of any bacteria except coagulase-negative Staphylococcus epidermidis [12,32]. For TLR2, TLR4, TLR6 and TLR9 receptors, their participation in angiogenesis, one of the key processes involved in the development and function of the placenta, has also been confirmed [14,33,34,35]. TLR2 has been demonstrated to induce the expression of the angiogenic factor angiopoietin 2 (ANGPT2), as well as the MMP2 and MMP9 proteins, involved in destabilization and development of blood vessels [36]. The macrophage-activating lipopeptide-2 (MALP2), isolated from Mycoplasma spp., was found to interact with TLR2/6 receptors to induce GM-CSF-mediated angiogenesis [14,37]. TLR9 has been shown to correlate with the inhibition of angiogenesis by downregulating the vascular endothelial growth factor (VEGF) and upregulating the soluble VEGF receptor 1 (sVEGFR1, sFLT1) at the fetomaternal interface with preeclampsia [33]. In addition, TLR9 has also been reported to be associated with increased mRNA and protein levels of TF but with a decreased transcription, secretion and activity of TFPI in human coronary artery endothelial cells and with activation of blood clotting in mice [38].

Given the genetic alterations in angiogenic factors, *CSF2* rs25881, rs25882 and rs27438 were associated with an increased risk of preterm birth in European-American women, while rs721121, rs4705916, rs743564 and rs6898270 were correlated with a reduced risk of the disease in both European and African-American pregnant women [39]. For *FLT1*, rs748252 was reported to be involved in an approximately two-fold increased risk of spontaneous preterm labor in women with bacterial vaginosis prior to the 37th week of gestation [40]. Among African-American women, a minor A allele in *FLT1* rs12428494 was associated with spontaneous preterm delivery, determined before the 34th week of pregnancy [41]. In turn, it was found that the *FLT1* rs722503 polymorphism correlated with an increased susceptibility to preeclampsia in a dominant model, designed with regards to Iranian women [42]. The T allele in rs722503 was significantly more prevalent among women with preeclampsia, compared to healthy controls [42]. A study conducted on placentas with intrauterine growth restriction (IUGR) demonstrated an involvement of TFPI in alternative splicing and angiogenesis-related processes [43]. However, it was found that the frequency of genotypes and alleles for *TFPI* rs8176592 was similar, both in women with recurrent miscarriages and in healthy controls [44]. The genotype prevalence rates for *TFPI* T-33C and C-399T polymorphisms were also similar, both in cases with recurrent pregnancy loss and in the control group, while the *TFPI* -287C allele (TC + CC genotypes) was considered to protect against the disease [45]. In the case of the *TLR9* gene, it was reported that the CT and TT genotypes within rs352140 were significantly more common in neonates with placental inflammation compared to the CC genotype [46]. So far, it has been shown that chorioamnionitis is associated with preterm labor and related postpartum diseases [46]. In Ukrainian pregnant women, *TLR9* rs352140 has been reported to play a key role in the development of spontaneous abortion [47]. The AA and GA genotypes, as well as the A allele in rs352140, significantly increased the risk of miscarriage [47]. Among Ghanaian primiparas infected with Plasmodium falciparum, *TLR9* rs187084 was associated with a six-fold increased risk of low birth weight in full-term infants but was not involved in preterm delivery [48].

Since there is a lack of data on the contribution of genetic changes associated with hemostasis and angiogenesis to the occurrence of PROM, the current research aimed to demonstrate the role of the four SNPs, located in *CSF2* (rs25881), *FLT1* (rs722503), *TFPI* (C-399T) and *TLR9* (rs352140) genes, in the presented disease.

## 2. Materials and Methods

### 2.1. Women with PROM and Healthy Controls

The study was conducted prospectively in 360 women with singleton pregnancy, treated at the Department of Obstetrics, Perinatology and Gynecology of the Polish Mother’s Memorial Hospital-Research Institute (PMMH-RI), in Lodz, Poland, during the period between August 2016 and December 2020 (see Table 1). The population consisted of 180 women with PROM, observed between 14 and 41 weeks of gestation, and 180 healthy full-term women without PROM as controls. In the studied cohort, 126 (70.0%) women were diagnosed with pPROM before the 37th week of pregnancy, while 54 (30.0%) women with tPROM from 37 weeks of gestation (see Table 1 and Table S1). The presence of PROM was confirmed by the AMNIOQUICK^®^ test (BIOSYNEX SA, Illkirch-Graffenstaden, France). The pregnant women included in the study were aged between 18 and 44 years, the PROMs were aged between 18 and 43 years, and the controls were aged between 18 and 44 years. The women with pPROM ranged in age from 18 to 43 years, while those with tPROM ranged from 19 to 37 years in age. Detailed clinical characteristics of the women in terms of asthma and respiratory system infections, bleeding, diabetes mellitus (DM), hypertension, hypothyroidism, serological conflict, threatened miscarriage and genitourinary infections, are presented in Table 1 and Table S1. Women were excluded from the study in the following cases: multiple pregnancy, congenital disorder, genetic syndrome, structural uterine defect, endometriosis, myoma uterus, placenta previa, cervical insufficiency, condition after amniocentesis, fetal abnormality and growth disorders, including fetal growth restriction (FGR), small for gestational age (SGA) and large for gestational age (LGA). The study was approved by the Research Ethics Committee at the PMMH-RI (approval numbers 14/2019 and 15/2019). Clinical samples were obtained for diagnostic purposes, then anonymized for research. Informed consent forms were signed by all the study participants, as recommended by the Research Ethics Committee.

### 2.2. Collection and Analysis of Blood Samples

Two S-Monovette tubes (Sarstedt, Numbrecht, Germany) were filled with peripheral venous blood, collected by puncture from each woman on the day of admission for research purposes. EDTA KE/1.2 mL tubes were used for complete blood count (CBC) and DNA extraction, while 9 NC/1.4 mL coagulation tubes were used to determine the activated partial thromboplastin time (APTT). Platelet (PLT) parameters, including PLT count, platelet distribution width (PDW), the mean platelet volume (MPV) and plateletcrit (PCT), as part of the CBC, were determined using Fluorocell PLT reagent on a Sysmex XN-2000 Automated Hematology System (Sysmex, Kobe, Japan). The PLT count is normal between 150 × 10^9^/L and 400 × 10^9^/L, and the MPV reference range is 8.0 to 10.0 fL, according to the manufacturer (Sysmex, Kobe, Japan). APTT was assessed using the HemosIL APTT-SP reagent on an ACL TOP 550 CTS automated system (Instrumentation Laboratory, Werfen Company, Bedford, MA, USA). The normal range of APTT is 23 to 36.9 s, as reported by the manufacturer. Total DNA was extracted from 200 μL of whole-blood samples using the Syngen Blood/Cell DNA Mini Kit (Syngen Biotech, Wroclaw, Poland). Purified DNA was eluted from a mini spin column in 100 μL of buffer DE and stored at −20 °C until further analysis.

### 2.3. Genotyping of Single Nucleotide Polymorphisms (SNPs)

**Figure 1:**
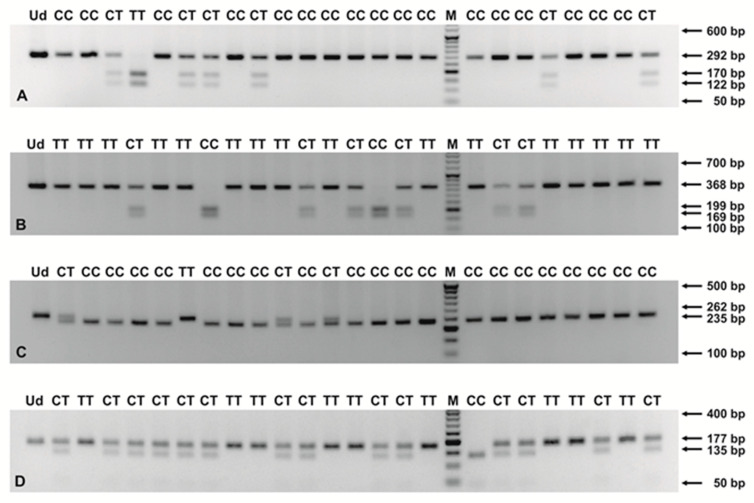
Products of PCR-RFLP assays for the *CSF2* rs25881 (**A**), *FLT1* rs722503 (**B**), *TFPI* C-399T (**C**) and *TLR9* rs352140 (**D**) polymorphisms. Digestions were performed with the endonucleases BlpI (**A**), AvaII (**B**), HinfI (**C**) and BstUI (**D**), followed by separation in 2.5–3.0% ethidium bromide stained agarose gels. The numbers to the right of the electropherograms show the lengths of the separated DNA fragments. M: 50 bp DNA marker; Ud: undigested PCR product; CC, CT, TT: genotypes in the tested SNPs.

Four single nucleotide polymorphisms (SNPs) from *CSF2* (rs25881), *FLT1* (rs722503), *TFPI* (C-399T) and *TLR9* (rs352140) genes were genotyped by the polymerase chain reaction-restriction fragment length polymorphism (PCR-RFLP) method. SNPs were selected for the study, taking into account (1) their localization in the genes, associated with angiogenesis and PROM, and (2) their possible influence on protein function. For the three genes, i.e., *CSF2*, *FLT1* and *TLR9*, SNPs were found from the National Center for Biotechnology Information (NCBI) SNP database (dbSNP) (https://www.ncbi.nlm.nih.gov/snp/ accessed on 22 September 2021) [49]. The minor allele frequency (MAF) of rs25881, rs722503 and rs352140 was >10%, as reported by the NCBI Allele Frequency Aggregator (ALFA) project. Primer sequences, restriction enzymes and RFLP profiles are shown in Table S2, as previously described [42,49,50,51]. The PCR mixes, with a final volume of 25 μL, contained up to 0.5 μg of total DNA, 0.2 mM dNTP mix, 0.4 μM of each primer, specific for the polymorphism to be evaluated, 1x polymerase B buffer and 0.5 U of Perpetual Taq DNA Polymerase (EURx, Gdańsk, Poland). The PCR program included initial denaturation at 95 °C for 3 min, 40 cycles of denaturation at 95 °C for 30 s, annealing at 55–58 °C, depending on SNP for 40 s, extension at 72 °C for 1 min and final extension at 72 °C for 7 min. The amplicons were digested with 10 U of the appropriate enzymes at defined temperatures for 16 h. PCR and restriction digestions were performed on a T100 Thermal Cycler (Bio-Rad, Singapore). The PCR and RFLP products were separated in 1–3.0% agarose gels (see Figure 1), prepared in 1× TAE buffer, depending on the length of the tested DNA fragments, and visualized in the ChemiDoc XRS+ imaging system (Bio-Rad, Hercules, CA, USA).

### 2.4. Statistical Analysis

The study groups of the women with PROM and healthy controls and their offspring were characterized by descriptive statistics and then compared using Pearson’s chi-square and Mann–Whitney U tests. The female clinical data and the APTT and PLT parameters were described and compared among groups using the NCSS 2004 software. The Hardy–Weinberg equilibrium and the prevalence rates of alleles, genotypes and multiple-SNP variants for *CSF2*, *FLT1*, *TLR9* and *TFPI* polymorphisms were determined using the SNPStats software [52]. The allele distribution among the examined groups of women was estimated using Pearson’s chi-square test. The relationships between genetic changes and the prevalence of PROM were estimated using logistic regression analyses to define inheritance models. Multiple-SNP analyses were performed using the expectation–maximization (EM) algorithm to estimate the frequency of complex variants in the PROM and healthy control groups. Adjustment analyses of the results for the APTT, PLT parameters and pregnancy disorders, including asthma and respiratory infections, bleeding, DM, hypertension, hypothyroidism, serological conflict, threatened miscarriage and urogenital infections, were also performed using logistic regression models. The results were considered statistically significant at the significance level of *p* ≤ 0.050.

## 3. Results

### 3.1. Characteristics of the Pregnant Women

The women with PROM and the healthy controls were of similar age (*p* = 0.057, see Table 1). The women with pPROM were significantly older compared to both the control group and the women with tPROM (*p* ≤ 0.001, see Table 1 and Table S1). Many pregnancies occurred significantly more frequently in PROM cases than in the control group (*p* ≤ 0.050, see Table 1). Women in more than one pregnancy demonstrated pPROM more often than tPROM (*p* ≤ 0.001, see Table S1). The methods of delivery, including natural labor and caesarean section, were similarly distributed in the study groups (*p* = 0.891 and *p* = 0.419, see Table 1). The gestational age at delivery was significantly shorter in women with PROM than in those in the control group (*p* ≤ 0.001). As for the offspring of the women, male births were more common in the women with PROM, compared to healthy controls (*p* = 0.045). In addition, the fetal weight and Apgar at 1 and 5 min were significantly lower in the PROM cases than in the control group (*p* ≤ 0.050). A comparison between pPROM and tPROM showed similar fetal weight (*p* = 0.240, see Table S1), but significantly lower Apgar score (after 1 and 5 min) was found in the women with pPROM (*p* ≤ 0.001).

### 3.2. Parameters of Hemostasis

APTT and PCT achieved similar values in the studied groups of pregnant women (*p* > 0.050, see Table 1 and Table S1). The PLT count was comparable between PROMs and controls; however, the count was significantly higher in women with pPROM than in those with tPROM (*p* = 0.028). In turn, PDW and MPV were similar in cases of PROM and healthy controls, but they reached lower values in the pPROM women when compared to the control group and the women with tPROM (*p* ≤ 0.050).

### 3.3. Hardy–Weinberg Equilibrium

For all tested groups of pregnant women, the Hardy–Weinberg (H–W) equilibrium was maintained for the genotypes in *FLT1* rs722503 and *TFPI* C-399T SNPs (*p* > 0.050). In the case of *CSF2* rs25881, the H–W equilibrium was found in the women with pPROM and in the healthy control group, but it was not confirmed in the tPROM cases (*p* = 0.027). Regarding *TLR9* rs352140, genotypes were found in the H–W equilibrium in the women with tPROM and in the healthy controls, while the deviation was significant in the pPROM women (*p* ≤ 0.001).

### 3.4. Genetic Alterations in CSF2, FLT1, TFPI and TLR9 Polymorphisms

A single-SNP analysis provided similar prevalence rates of genotypes in *CSF2* rs25881, *FLT1* rs722503, *TFPI* C-399T and *TLR9* rs352140 SNPs among the women with PROM or pPROM and the healthy controls (see Table S3a,b). Similar prevalence rates of genotypes for the studied polymorphisms were also found in the pPROM and tPROM cases (see Tables S4–S7). Alleles in the tested SNPs had a similar distribution pattern among the studied groups of pregnant women (see Tables S5 and S6). However, the CT heterozygotes in the *CSF2* polymorphism were significantly more frequent among the women with pPROM when compared to those with tPROM after correction by DM (OR 2.28 95% CI 1.04–5.01, *p* = 0.032, see Table 2).

A multiple-SNP analysis showed that the TT variants for *CSF2* and *FLT1* polymorphisms correlated with an approximately two-fold increase in the PROM risk when corrected for APTT, PLT parameters and pregnancy disorders (*p* ≤ 0.050, see Table 3 and Table S7). CC double-SNP variants for *TLR9* and *TFPI* polymorphisms were also associated with an almost two-fold higher risk of PROM, corrected by APTT (OR 1.94 95% CI 1.08–3.50, *p* = 0.028, see Table 3). Triple-SNP variants of TTC for the *CSF2*, *FLT1* and *TLR9* polymorphisms were associated with an increased risk of PROM when adjusted for APTT (OR 19.54 95% CI 1.22–311.80, *p* = 0.037). In turn, TCT variants for those three SNPs correlated with a reduced risk of PROM after adjusting for PLT (OR 0.13 95% CI 0.13–0.14, *p* ≤ 0.001). Similarly, CCTC variants for *CSF2*, *FLT1*, *TLR9* and *TFPI* SNPs were significantly less frequent among the PROMs than the healthy controls, corrected by APTT (OR 0.04 95% CI 0.01–0.28, *p* = 0.002). It was found that TTT triple-SNP variants for *FLT1*, *TLR9* and *TFPI* polymorphisms, as well as CCCC and TTTC complex variants for all the tested SNPs, were correlated with an increased risk of PROM (*p* ≤ 0.050).

A comparison between the women with pPROM and the healthy controls showed that TT double-SNP variants for *CSF2* and *FLT1* polymorphisms and TTT triple-SNP variants for *CSF2*, *FLT1* and *TLR9* polymorphisms correlated with an increased risk of disease when corrected by APTT and PLT parameters (*p* ≤ 0.050, see Tables S8–S10). Adjusting the results for pregnancy disorders also revealed that the TT variants for *CSF2* and *FLT1* SNPs were associated with an approximately two-fold increase in the pPROM risk (*p* ≤ 0.050, see Table 4), while the TCT complex variants for the *CSF2*, *FLT1* and *TLR9* SNPs correlated with a reduced risk of disease, corrected by a serological conflict (OR 0.01 95% CI 0.00–0.05, *p* ≤ 0.001, see Table 4). The complex TCC and TTT variants for the *FLT1*, *TLR9* and *TFPI* SNPs were correlated with a significantly higher risk of pPROM when considering the APTT and PLT parameters (*p* ≤ 0.050). In turn, triple-SNP CCC variants for those three polymorphisms were associated with a significantly lower risk of pPROM after adjusting to APTT (OR 0.04 95% CI 0.00–0.66, *p* = 0.026). The CCC variants for *CSF2*, *FLT1* and *TFPI* polymorphisms were also associated with an increased risk of pPROM after adjusting for PLT parameters (OR 1.65 95% CI 1.03–2.64, *p* = 0.036).

Moreover, quadruple-SNP variants CTTT and TTTC for *CSF2*, *FLT1*, *TLR9* and *TFPI* polymorphisms were also found to correlate with a higher risk of pPROM when adjusted for PLT parameters.

A further analysis showed that different double-, triple- and quadruple-SNP variants for the studied polymorphisms with C alleles in *CSF2* rs25881, *FLT1* rs722503, *TFPI* C-399T SNPs and the T allele in *TLR9* rs352140 polymorphism were significantly more frequent in the women with pPROM compared to the PROM subjects, after adjusting for APTT, PLT parameters and pregnancy disorders (*p* ≤ 0.050, see Tables S9 and S10). The complex TTTT variants for all the tested SNPs were more common in the pPROM women than in those with tPROM, corrected by PLT and PDW (OR 6.05 95% CI 4.71–7.78, *p* ≤ 0.001).

## 4. Discussion

We found in our study that genetic changes, localized in SNPs from *CSF2*, *FLT1*, *TFPI* and *TLR9* genes, were associated with PROM, when the results had been adjusted for APTT, PLT parameters or pregnancy disorders. Among the double-SNP variants, the TT complex genotypes for *CSF2* rs25881 and *FLT1* rs722503 polymorphisms correlated with an increased risk of PROM and pPROM, after adjusting for APTT, PLT parameters or pregnancy disorders. In women of European and American origin, *CSF2* rs25881 was found to be associated with preterm birth; however, no predelivery events were defined, such as preterm labor or spontaneous rupture of membranes [39]. Regarding *FLT1* rs722503, the T allele was previously reported to be associated with an increased risk of preeclampsia in populations of Iranian and white pregnant women [42,53]. Similarly, AA homozygotes in rs722503 were estimated to be susceptible to preeclampsia in women from the Philippines [54]. We also found that the CC double-SNP variants for the *TLR9* rs352140 and *TFPI* C-399T SNPs were associated with an approximately two-fold higher risk of PROM when the results were corrected by APTT. In patients with stable coronary heart disease, the T allele in the *TFPI* C-399T polymorphism was correlated with an increased thrombin generation in vivo, and TT homozygotes were associated with an extended ex vivo thrombin generation delay time [55]. Moreover, in cultured human coronary artery endothelial cells, TLR9 was found to shift the TF and TFPI balance towards a procoagulant phenotype when induced by bacterial DNA [38]. In the case of TF, increased mRNA and protein levels and activity were determined, while *TFPI* had lower transcription, secretion and activity [38]. Considering our results, a combined participation of the tested SNPs from *TLR9* and *TFPI* genes, involved in hemostasis in PROM, seems likely.

Regarding the coagulation parameters, it was previously shown that prothrombin time (PT) and APTT were significantly shorter in preterm labor [56]. In the case of PLT parameters, the PLT count, as well as MPV and PCT, were also shown as correlating with PROM [57]. Among women with pPROM in the first trimester, a significantly higher PLT count and reduced MPV values were determined compared to healthy controls [57]. In our cohort of women, we also found significantly lower MPV values in the pPROM cases than in the healthy controls. Therefore, it seems important to adjust the genetic results of this study to the APTT and PLT parameters. To date, MPV reduction has been associated with chronic inflammatory disorders, including inflammatory bowel disease, rheumatoid arthritis, acute rheumatic fever and ankylosing spondylitis [58].

Taking into account triple-SNP variants for *CSF2*, *FLT1* and *TLR9* polymorphisms, we found that TTC variants correlated with an approximately 20-fold increased risk of PROM when adjusted by APTT, while TCT variants were associated with a decreased risk of the disease after correction by the PLT count. Among the pPROM cases, the TTT complex variants were significantly more prevalent compared to the healthy controls when the results were adjusted for PLT parameters. Conversely, TCT variants were correlated with a reduced risk of pPROM after adjusting for serological conflict. Regarding *TLR9* rs352140, CT and/or TT genotypes were previously found to be involved in placental inflammation and maternal pattern of inflammation [46]. Another study, performed in the Polish population, showed *TLR9* rs352140 as a possible genetic risk factor for cervical cancer [59]. In terms of our results, it also seems possible that *CSF2*, *FLT1* and *TLR9* SNPs collaborate in the development of PROM. It is noteworthy that TLR9 has been reported to inhibit angiogenesis by downregulating VEGFA and upregulating sFLT1 in placentas from an animal model of preeclampsia and in trophoblasts [33]. Similarly, the TLR9 ligand, oligodeoxynucleotide (ODN) 1826, has been found to induce sFLT1 secretion from macrophages and decrease the number of aortic ring vessel sprouts [60]. In the suture-induced corneal angiogenesis model, ODN 1826 has also been determined to reduce the length and volume of hemangiogenesis and lymphangiogenesis [60]. We found in our study that TTT triple-SNP variants for *FLT1*, *TLR9* and *TFPI* polymorphisms were associated with an increased risk of PROM and pPROM when adjusted by APTT or PLT parameters. Similarly, TCC variants were more common in the women with pPROM than in the healthy controls, while CCC complex variants correlated with a decreased risk of the disease when corrected by APTT. Taking into account the results, obtained also by the quadruple-SNP analysis, the participation of the four studied polymorphisms in PROM seems justified. An additional comparison between the pPROM and tPROM cases showed that the C alleles for the *CSF2*, *FLT1* and *TFPI* polymorphisms, and the T allele for the *TLR9* SNP in different complex variants, were more common in the women with pPROM after adjusting for APTT, PLT parameters and pregnancy disorders.

In this study, we corrected the results for the following complications of pregnancy: asthma and respiratory infections, bleeding, DM, hypertension, hypothyroidism, serological conflict, threatened miscarriage and urogenital infections. So far, many studies have described the relationship between these diseases and the occurrence of PROM [61,62,63,64,65]. DM-adjusted analyses showed that *CSF2* rs25881 CT heterozygotes are significantly more common in pPROM compared to tPROM cases. Similarly, the TT double-SNP variants for the *CSF2* and *FLT1* polymorphisms had an approximately two-fold increased risk of PROM when the results were adjusted for DM and other pregnancy disorders. Previously, pre-pregnancy DM, as well as nulliparity, maternal age and body mass index (BMI), were termed the predictors of pPROM [66]. In another study, gestational DM (GDM) was associated with an increased incidence of vulvovaginal candidiasis, PROM, preterm labor, chorioamnionitis/puerperal infection, as well as macrosomia, and neonatal hypoglycemia [67]. Asthma was found to be positively associated with PROM, both at preterm and term pregnancies, while chronic bronchitis correlated with a reduced risk of the disease [68]. In turn, vaginal bleeding has been shown to correlate with a shorter gestational age at membrane rupture and delivery, as well as lower birth weight, more frequent placental abruption, RDS, IVH and perinatal death [69]. Considering hypertensive disorders, gestational hypertension was found to be associated with an approximately four-fold increased risk of PROM, while preeclampsia was correlated with about two-fold higher risk [70]. Moreover, hypertension was associated with an increased risk of term rather than preterm PROM [70]. In turn, hypothyroidism in pregnancy was correlated with a slight FGR, a higher risk of PROM and the development of hypertension and GDM [62]. Similarly, threatened miscarriage was associated with much more frequent PROM, preterm labor and low-birth-weight newborns [71]. PPROM was found more prevalent in women with threatened miscarriage and in a high-risk group with the risk factor for spontaneous abortion compared to healthy controls [72]. Another significant risk factor for subsequent pPROM turned out to be the composition of the vaginal microbiota, and vaginal dysbiosis was correlated with unfavorable short-term outcomes of mothers and newborns [73,74]. Therefore, it is important to correct the obtained genetic results for the presented study population of pregnant women for possible risk factors of PROM.

## 5. Conclusions

Polymorphisms of the genes involved in hemostasis and angiogenesis, including *CSF2* rs25881, *FLT1* rs722503, *TFPI* C-399T and *TLR9* rs352140, contribute to PROM.

## Figures and Tables

**Table 1:** Characteristics of women with prelabor rupture of membranes and healthy controls.

		Controls	PROM ^a^ Cases	*p*-Value ^b^	pPROM ^c^ Cases	*p*-Value
**Number of women**		180	180		126	
**Age (years)**		28 (18–44)	30 (18–43)	0.057	31 (18–43)	**≤0.001**
**No. ^d^ of pregnancy, *n* (%)**	**1**	113 (62.8%)	87 (48.3%)	**0.013**	47 (37.3%)	**≤0.001**
	**2**	51 (28.3%)	55 (30.6%)		45 (35.7%)	
	**3**	12 (6.7%)	26 (14.4%)		22 (17.5%)	
	**4**	4 (2.2%)	7 (3.9%)		7 (5.5%)	
	**5**	0 (0.0%)	3 (1.7%)		3 (2.4%)	
	**6**	0 (0.0%)	2 (1.1%)		2 (1.6%)	
**Pregnancy disorders, *n* (%)**	**Asthma and respiratory system infections**	4 (2.2%)	10 (5.6%)	0.085	6 (4.8%)	0.183
	**Bleeding**	3 (1.7%)	7 (3.9%)	0.168	5 (4.0%)	0.189
	**Diabetes mellitus**	28 (15.6%)	18 (10.0%)	0.114	16 (12.7%)	0.483
	**Hypertension**	22 (12.2%)	19 (10.6%)	0.619	13 (10.3%)	0.606
	**Hypothyroidism**	27 (15.0%)	36 (20.0%)	0.212	25 (19.8%)	0.267
	**Serological conflict**	11 (6.1%)	4 (2.2%)	0.055	2 (1.6%)	**0.045**
	**Threatened miscarriage**	0 (0.0%)	15 (8.3%)	**≤0.001**	12 (9.5%)	**≤0.001**
	**Urogenital infections**	20 (11.1%)	20 (11.1%)	1.000	11 (8.7%)	0.497
**APTT (s) ^e^**		28.3 (23.6–34.8)	27.7 (21.7–44.5)	0.103	28.1 (21.7–44.5)	0.322
**Platelet parameters**	**No. [×10^9^/L]**	211.5 (150–398)	214 (59–457)	0.220	220.5 (59–457)	0.900
	**PDW [fL] ^f^**	13.7 (9.0–23.7)	13.3 (8.8–24.9)	0.597	12.9 (8.8–24.9)	**0.010**
	**MPV [fL] ^g^**	11.1 (9.0–14.1)	11.1 (8.8–14.6)	0.809	10.9 (8.8–14.6)	**0.024**
	**PCT [%] ^h^**	0.24 (0.17–0.39)	0.23 (0.06–0.50)	0.086	0.23 (0.06–0.50)	0.257
**Delivery, *n* (%)**	**Weeks of pregnancy**	39 (37–41)	35 (17–41)	**≤0.001**	33 (17–40)	**≤0.001**
	**Natural**	82 (45.6%)	78 (44.8%)	0.891	49 (40.8%)	0.419
	**C-section ^i^**	98 (54.4%)	96 (55.2%)		71 (59.2%)	
**Fetal sex, *n* (%)**	**Female**	96 (53.3%)	72 (42.6%)	**0.045**	50 (43.5%)	0.099
	**Male**	84 (46.7%)	97 (57.4%)		65 (56.5%)	
**Neonatal data**	**Weight (percentiles)**	73 (10–100)	63.5 (0–100)	**0.004**	56.5 (0–100)	**0.002**
	**Apgar in 1 min**	10 (7–10)	9 (0–10)	**≤0.001**	7 (0–10)	**≤0.001**
	**Apgar in 5 min**	10 (7–10)	9 (0–10)	**≤0.001**	8 (0–10)	**≤0.001**

^a^ PROM, prelabor rupture of membranes; ^b^ *p*-value, statistically significant results are marked in bold; ^c^ pPROM, preterm PROM; ^d^ No., number; ^e^ APTT (s), activated partial thromboplastin time (second); ^f^ PDW, platelet distribution width; ^g^ MPV, mean platelet volume; ^h^ PCT, plateletcrit; ^i^ C-section, caesarean section.

**Table 2:** Distribution of genotypes in *CSF2*, *FLT1*, *TFPI* and *TLR9* SNPs between women with term and preterm PROM, adjusted for diabetes mellitus.

Polymorphism	Genetic Model	Genotype	Genotype Prevalence, *n* ^a^ (%)		OR ^d^ (95% CI ^e^)	*p*-Value ^f^
			tPROM ^b^	pPROM ^c^		
***CSF2***	Codominant	CC	40 (74.1%)	82 (65.1%)	1.00	0.055
**rs25881**		CT	10 (18.5%)	41 (32.5%)	2.16 (0.97–4.78)	
		TT	4 (7.4%)	3 (2.4%)	0.42 (0.09–1.96)	
	Dominant	CC	40 (74.1%)	82 (65.1%)	1.00	0.160
		CT-TT	14 (25.9%)	44 (34.9%)	1.66 (0.81–3.41)	
	Recessive	CC-CT	50 (92.6%)	123 (97.6%)	1.00	0.160
		TT	4 (7.4%)	3 (2.4%)	0.34 (0.07–1.56)	
	Overdominant	CC-TT	44 (81.5%)	85 (67.5%)	1.00	**0.032**
		CT	10 (18.5%)	41 (32.5%)	2.28 (1.04–5.01)	
***FLT1***	Codominant	TT	36 (66.7%)	71 (56.4%)	1.00	0.370
**rs722503**		CT	16 (29.6%)	48 (38.1%)	1.59 (0.79–3.20)	
		CC	2 (3.7%)	7 (5.6%)	1.80 (0.35–9.23)	
	Dominant	TT	36 (66.7%)	71 (56.4%)	1.00	0.160
		CT-CC	18 (33.3%)	55 (43.6%)	1.61 (0.82–3.16)	
	Recessive	TT-CT	52 (96.3%)	119 (94.4%)	1.00	0.600
		CC	2 (3.7%)	7 (5.6%)	1.52 (0.30–7.68)	
	Overdominant	TT-CC	38 (70.4%)	78 (61.9%)	1.00	0.230
		CT	16 (29.6%)	48 (38.1%)	1.52 (0.76–3.05)	
***TFPI***	Codominant	CC	43 (79.6%)	103 (81.8%)	1.00	0.530
**C-399T**		CT	11 (20.4%)	22 (17.5%)	0.74 (0.33–1.70)	
		TT	0 (0%)	1 (0.8%)	NA ^g^ (0.00–NA)	
	Dominant	CC	43 (79.6%)	103 (81.8%)	1.00	0.570
		CT-TT	11 (20.4%)	23 (18.2%)	0.79 (0.35–1.78)	
	Recessive	CC-CT	54 (100%)	125 (99.2%)	1.00	0.380
		TT	0 (0%)	1 (0.8%)	NA (0.00–NA)	
	Overdominant	CC-TT	43 (79.6%)	104 (82.5%)	1.00	0.470
		CT	11 (20.4%)	22 (17.5%)	0.74 (0.32–1.69)	
***TLR9***	Codominant	TT	13 (24.1%)	33 (26.2%)	1.00	0.510
**rs352140**		CT	32 (59.3%)	81 (64.3%)	0.88 (0.41–1.90)	
		CC	9 (16.7%)	12 (9.5%)	0.54 (0.18–1.57)	
	Dominant	TT	13 (24.1%)	33 (26.2%)	1.00	0.560
		CT-CC	41 (75.9%)	93 (73.8%)	0.80 (0.38–1.69)	
	Recessive	TT-CT	45 (83.3%)	114 (90.5%)	1.00	0.270
		CC	9 (16.7%)	12 (9.5%)	0.59 (0.23–1.49)	
	Overdominant	TT-CC	22 (40.7%)	45 (35.7%)	1.00	0.810
		CT	32 (59.3%)	81 (64.3%)	1.08 (0.56–2.11)	

^a^*n*, number; ^b^ tPROM, term prelabor rupture of membranes; ^c^ pPROM, preterm PROM; ^d^ OR, odds ratio; ^e^ 95% CI, confidence interval; ^f^ *p*-value, statistically significant result is marked in bold; ^g^ NA, not analyzed.

**Table 3:** Association of multiple-SNP variants for *CSF2*, *FLT1*, *TLR9* and *TFPI* polymorphisms with PROM after correction by APTT and PLT parameters.

Categorical Covariate	Polymorphisms/Alleles				Multiple-SNP ^a^ Variant Frequency		OR ^c^ (95% CI ^d^)	*p*-Value ^e^
	*CSF2*	*FLT1*	*TLR9*	*TFPI*	Controls	PROM ^b^ Cases		
	rs25881	rs722503	rs352140	C-399T				
**APTT ^f^**	C	T	-	-	0.629	0.592	1.00	---
	C	C	-	-	0.199	0.228	0.99 (0.53–1.86)	0.990
	T	T	-	-	0.118	0.181	2.39 (1.23–4.64)	**0.011**
	T	C	-	-	0.054	0.000	0.00 (−Inf ^k^–Inf)	1.000
	C	T	T	-	0.356	0.318	1.00	---
	C	T	C	-	0.275	0.273	1.46 (0.67–3.16)	0.340
	C	C	T	-	0.134	0.122	0.74 (0.23–2.40)	0.610
	T	T	T	-	0.079	0.129	1.96 (0.80–4.83)	0.150
	C	C	C	-	0.064	0.106	3.17 (0.63–15.81)	0.160
	T	T	C	-	0.038	0.051	19.54 (1.22–311.80)	**0.037**
	T	C	C	-	0.026	0.000	0.00 (−Inf–Inf)	1.000
	-	-	T	C	0.555	0.503	1.00	---
	-	-	C	C	0.364	0.400	1.94 (1.08–3.50)	**0.028**
	-	-	T	T	0.039	0.066	2.88 (0.50–16.61)	0.240
	-	-	C	T	0.041	0.031	0.80 (0.08–8.42)	0.860
	C	T	T	C	0.326	0.269	1.00	---
	C	T	C	C	0.244	0.255	1.03 (0.44–2.46)	0.940
	C	C	T	C	0.125	0.115	0.04 (0.01–0.28)	**0.002**
	T	T	T	C	0.080	0.118	0.41 (0.10–1.67)	0.210
	C	C	C	C	0.053	0.094	13.87 (1.96–98.21)	**0.009**
	T	T	C	C	0.036	0.052	35.00 (3.14–390.81)	**0.004**
	C	T	T	T	0.029	0.056	1.60 (0.27–9.60)	0.610
	T	C	C	C	0.032	0.011	0.09 (0.00–2.29)	0.140
	T	C	T	C	0.026	NA ^l^	1.17 (0.20–6.86)	0.860
	C	T	C	T	0.030	NA	0.01 (0.00–1.30)	0.066
	C	C	C	T	0.010	0.019	2.67 (0.11–66.45)	0.550
**PLT ^g^**	C	T	-	-	0.629	0.592	1.00	---
	C	C	-	-	0.199	0.228	1.15 (0.79–1.69)	0.460
	T	T	-	-	0.118	0.181	1.57 (1.01–2.43)	**0.045**
	T	C	-	-	0.054	0.000	0.00 (−Inf–Inf)	1.000
	C	T	T	-	0.356	0.318	1.00	---
	C	T	C	-	0.275	0.273	1.17 (0.70–1.96)	0.540
	C	C	T	-	0.134	0.122	1.01 (0.52–1.96)	0.980
	T	T	T	-	0.079	0.129	1.67 (0.88–3.16)	0.120
	C	C	C	-	0.064	0.106	1.61 (0.79–3.30)	0.190
	T	T	C	-	0.038	0.051	1.66 (0.61–4.49)	0.320
	T	C	T	-	0.026	0.000	0.13 (0.13–0.14)	**≤0.001**
	T	C	C	-	0.030	0.000	0.00 (−Inf–Inf)	1.000
	-	T	T	C	0.405	0.385	1.00	---
	-	T	C	C	0.280	0.309	1.24 (0.78–1.98)	0.370
	-	C	T	C	0.150	0.119	0.84 (0.46–1.54)	0.570
	-	C	C	C	0.084	0.090	1.11 (0.57–2.17)	0.770
	-	T	T	T	0.029	0.066	2.75 (1.05–7.19)	**0.040**
	-	T	C	T	0.033	0.012	0.00 (−Inf–Inf)	1.000
	-	C	C	T	0.008	0.019	2.42 (0.29–20.09)	0.410
	C	T	T	C	0.326	0.269	1.00	---
	C	T	C	C	0.244	0.255	1.50 (0.86–2.60)	0.150
	C	C	T	C	0.125	0.115	1.10 (0.53–2.30)	0.800
	T	T	T	C	0.080	0.118	1.81 (0.91–3.60)	0.094
	C	C	C	C	0.053	0.094	1.92 (0.87–4.27)	0.110
	C	T	T	T	0.029	0.056	2.70 (0.98–7.40)	0.055
	T	T	C	C	0.036	0.052	1.91 (0.67–5.40)	0.230
	T	C	C	C	0.026	NA	0.00 (−Inf–Inf)	1.000
	C	T	C	T	0.030	NA	0.00 (−Inf–Inf)	1.000
	T	C	T	C	0.032	0.011	0.31 (0.01–12.37)	0.530
	C	C	C	T	0.010	0.019	1.88 (0.29–12.08)	0.510
**PDW ^h^**	C	T	-	-	0.629	0.592	1.00	---
	C	C	-	-	0.199	0.228	1.22 (0.83–1.79)	0.320
	T	T	-	-	0.118	0.181	1.59 (1.03–2.46)	**0.039**
	T	C	-	-	0.054	0.000	0.00 (−Inf–Inf)	1.000
**MPV ^i^**	C	T	-	-	0.629	0.592	1.00	---
	C	C	-	-	0.199	0.228	1.22 (0.83–1.79)	0.320
	T	T	-	-	0.118	0.181	1.59 (1.03–2.46)	**0.038**
	T	C	-	-	0.054	0.000	0.00 (−Inf–Inf)	1.000
**PCT ^j^**	C	T	-	-	0.629	0.592	1.00	---
	C	C	-	-	0.199	0.228	1.20 (0.82–1.77)	0.350
	T	T	-	-	0.118	0.181	1.67 (1.07–2.61)	**0.024**
	T	C	-	-	0.054	0.000	0.00 (−Inf–Inf)	1.000
**PLT + MPV**	C	T	-	-	0.629	0.592	1.00	---
	C	C	-	-	0.199	0.228	1.20 (0.82–1.77)	0.350
	T	T	-	-	0.118	0.181	1.64 (1.06–2.56)	**0.028**
	T	C	-	-	0.054	0.000	0.00 (−Inf–Inf)	1.000
**PDW + MPV**	C	T	-	-	0.629	0.592	1.00	---
	C	C	-	-	0.199	0.228	1.21 (0.82–1.79)	0.340
	T	T	-	-	0.118	0.181	1.58 (1.02–2.45)	**0.041**
	T	C	-	-	0.054	0.000	0.00 (−Inf–Inf)	1.000
**MPV + PCT**	C	T	-	-	0.629	0.592	1.00	---
	C	C	-	-	0.199	0.228	1.21 (0.82–1.79)	0.340
	T	T	-	-	0.118	0.181	1.67 (1.07–2.61)	**0.024**
	T	C	-	-	0.054	0.000	0.00 (−Inf–Inf)	1.000
**PLT + PDW + MPV**	C	T	-	-	0.629	0.592	1.00	---
	C	C	-	-	0.199	0.228	1.20 (0.81–1.77)	0.370
	T	T	-	-	0.118	0.181	1.64 (1.05–2.55)	**0.030**
	T	C	-	-	0.054	0.000	0.00 (−Inf–Inf)	1.000
**PLT + PDW + PCT**	C	T	-	-	0.629	0.592	1.00	---
	C	C	-	-	0.199	0.228	1.24 (0.84–1.83)	0.280
	T	T	-	-	0.118	0.181	1.70 (1.09–2.66)	**0.019**
	T	C	-	-	0.054	0.000	0.00 (−Inf–Inf)	1.000
**PLT + MPV + PCT**	C	T	-	-	0.629	0.592	1.00	---
	C	C	-	-	0.199	0.228	1.24 (0.84–1.83)	0.280
	T	T	-	-	0.118	0.181	1.71 (1.09–2.66)	**0.019**
	T	C	-	-	0.054	0.000	0.00 (−Inf–Inf)	1.000
	C	T	T	C	0.326	0.269	1.00	---
	C	T	C	C	0.244	0.255	1.45 (0.82–2.56)	0.200
	C	C	T	C	0.125	0.115	1.26 (0.60–2.65)	0.540
	T	T	T	C	0.080	0.118	2.08 (1.02–4.23)	**0.045**
	C	C	C	C	0.053	0.094	2.00 (0.89–4.48)	0.092
	T	T	C	C	0.036	0.052	1.83 (0.65–5.15)	0.260
	C	T	T	T	0.029	0.056	2.55 (0.86–7.55)	0.091
**PDW + MPV + PCT**	C	T	-	-	0.629	0.592	1.00	---
	C	C	-	-	0.199	0.228	1.20 (0.81–1.78)	0.360
	T	T	-	-	0.118	0.181	1.66 (1.06–2.60)	**0.026**
	T	C	-	-	0.054	0.000	0.00 (−Inf–Inf)	1.000
**PLT + PDW + MPV + PCT**	C	T	-	-	0.629	0.592	1.00	---
	C	C	-	-	0.199	0.228	1.22 (0.83–1.81)	0.310
	T	T	-	-	0.118	0.181	1.69 (1.08–2.64)	**0.022**
	T	C	-	-	0.054	0.000	0.00 (−Inf–Inf)	1.000

^a^ SNP, single nucleotide polymorphism; ^b^ PROM, prelabor rupture of membranes; ^c^ OR, odds ratio; ^d^ 95% CI, confidence interval; ^e^ *p*-value, statistically significant results are marked in bold; ^f^ APTT, activated partial thromboplastin time; ^g^ PLT, platelet; ^h^ PDW, platelet distribution width; ^i^ MPV, mean platelet volume; ^j^ PCT, plateletcrit; ^k^ Inf, infinity; ^l^ NA, not analyzed.

**Table 4:** Relationship between multiple-SNP variants for *CSF2*, *FLT1* and *TLR9* polymorphisms and occurrence of preterm PROM after correction for pregnancy disorders.

Pregnancy Disorders	Polymorphisms/Alleles			Multiple-SNP ^a^ Variant Frequency		OR ^c^ (95% CI ^d^)	*p*-Value ^e^
	*CSF2*	*FLT1*	*TLR9*	Controls	pPROM ^b^ Cases		
	rs25881	rs722503	rs352140				
**Asthma and respiratory system infections**	C	T	-	0.629	0.568	1.00	---
	C	C	-	0.199	0.246	1.39 (0.92–2.09)	0.110
	T	T	-	0.118	0.187	1.72 (1.06–2.78)	**0.028**
	T	C	-	0.054	0.000	0.00 (−Inf ^f^–Inf)	1.000
	C	T	T	0.356	0.299	1.00	---
	C	T	C	0.275	0.276	1.36 (0.76–2.43)	0.300
	C	C	T	0.134	0.150	1.59 (0.77–3.28)	0.210
	T	T	T	0.079	0.134	1.98 (0.97–4.04)	0.063
	C	C	C	0.064	0.089	1.69 (0.73–3.93)	0.220
	T	T	C	0.038	0.045	2.09 (0.65–6.76)	0.220
	T	C	C	0.030	0.008	0.00 (−Inf–Inf)	1.000
	T	C	T	0.026	0.000	0.00 (−Inf–Inf)	1.000
**Bleeding**	C	T	-	0.629	0.568	1.00	---
	C	C	-	0.199	0.246	1.38 (0.92–2.07)	0.120
	T	T	-	0.118	0.187	1.73 (1.07–2.79)	**0.026**
	T	C	-	0.054	0.000	0.00 (−Inf–Inf)	1.000
	C	T	T	0.356	0.299	1.00	---
	C	T	C	0.275	0.276	1.27 (0.68–2.39)	0.450
	C	C	T	0.134	0.150	1.52 (0.73–3.14)	0.260
	T	T	T	0.079	0.134	2.00 (0.97–4.10)	0.060
	C	C	C	0.064	0.089	1.63 (0.67–3.95)	0.280
	T	T	C	0.038	0.045	1.80 (0.24–13.52)	0.570
	T	C	C	0.030	0.008	0.03 (0.00–3.29 × 10^26^)	0.920
	T	C	T	0.026	0.000	0.00 (−Inf–Inf)	1.000
**Diabetes mellitus**	C	T	-	0.629	0.568	1.00	---
	C	C	-	0.199	0.246	1.35 (0.90–2.02)	0.150
	T	T	-	0.118	0.187	1.72 (1.06–2.77)	**0.027**
	T	C	-	0.054	0.000	0.00 (−Inf–Inf)	1.000
	C	T	T	0.356	0.299	1.00	---
	C	T	C	0.275	0.276	1.29 (0.61–2.72)	0.510
	C	C	T	0.134	0.150	1.41 (0.52–3.77)	0.500
	T	T	T	0.079	0.134	1.94 (0.93–4.05)	0.080
	C	C	C	0.064	0.089	1.63 (0.50–5.34)	0.420
	T	T	C	0.038	0.045	1.73 (0.03–95.93)	0.790
	T	C	C	0.030	0.008	0.11 (0.00–8.01 × 10^15^)	0.910
	T	C	T	0.026	0.000	0.11 (0.00–7.18 × 10^11^)	0.880
**Hypertension**	C	T	-	0.629	0.568	1.00	---
	C	C	-	0.199	0.246	1.35 (0.90–2.03)	0.140
	T	T	-	0.118	0.187	1.71 (1.06–2.75)	**0.029**
	T	C	-	0.054	0.000	0.00 (−Inf–Inf)	1.000
	C	T	T	0.356	0.299	1.00	---
	C	T	C	0.275	0.276	1.26 (0.71–2.23)	0.440
	C	C	T	0.134	0.150	1.46 (0.71–2.99)	0.300
	T	T	T	0.079	0.134	1.92 (0.94–3.92)	0.074
	C	C	C	0.064	0.089	1.62 (0.70–3.75)	0.260
	T	T	C	0.038	0.045	1.90 (0.59–6.12)	0.280
	T	C	C	0.030	0.008	0.00 (−Inf–Inf)	1.000
	T	C	T	0.026	0.000	0.00 (−Inf–Inf)	1.000
**Hypothyroidism**	C	T	-	0.629	0.568	1.00	---
	C	C	-	0.199	0.246	1.36 (0.90–2.04)	0.140
	T	T	-	0.118	0.187	1.71 (1.06–2.77)	**0.029**
	T	C	-	0.054	0.000	0.00 (−Inf–Inf)	1.000
	C	T	T	0.356	0.299	1.00	---
	C	T	C	0.275	0.276	1.28 (0.72–2.26)	0.400
	C	C	T	0.134	0.150	1.47 (0.72–2.98)	0.290
	T	T	T	0.079	0.134	2.00 (0.98–4.10)	0.058
	C	C	C	0.064	0.089	1.65 (0.72–3.80)	0.240
	T	T	C	0.038	0.045	1.76 (0.53–5.79)	0.360
	T	C	C	0.030	0.008	0.01 (−Inf–Inf)	1.000
	T	C	T	0.026	0.000	0.00 (−Inf–Inf)	1.000
**Serological conflict**	C	T	-	0.629	0.568	1.00	---
	C	C	-	0.199	0.246	1.32 (0.88–1.98)	0.180
	T	T	-	0.118	0.187	1.66 (1.03–2.69)	**0.039**
	T	C	-	0.054	0.000	0.00 (−Inf–Inf)	1.000
	C	T	T	0.356	0.299	1.00	---
	C	T	C	0.275	0.276	1.27 (0.68–2.39)	0.450
	C	C	T	0.134	0.150	1.37 (0.65–2.86)	0.410
	T	T	T	0.079	0.134	1.93 (0.94–3.96)	0.076
	C	C	C	0.064	0.089	1.63 (0.64–4.14)	0.310
	T	T	C	0.038	0.045	1.53 (0.18–12.75)	0.690
	T	C	C	0.030	0.008	0.14 (0.00–1.44 × 10^6^)	0.810
	T	C	T	0.026	0.000	0.01 (0.00–0.05)	**≤0.001**
**Threatened miscarriage**	C	T	-	0.629	0.568	1.00	---
	C	C	-	0.199	0.246	1.34 (0.89–2.02)	0.170
	T	T	-	0.118	0.187	1.69 (1.03–2.77)	**0.037**
	T	C	-	0.054	0.000	0.00 (−Inf–Inf)	1.000
	C	T	T	0.356	0.299	1.00	---
	C	T	C	0.275	0.276	1.25 (0.70–2.23)	0.460
	C	C	T	0.134	0.150	1.54 (0.71–3.36)	0.280
	T	T	T	0.079	0.134	1.94 (0.90–4.21)	0.094
	C	C	C	0.064	0.089	1.34 (0.55–3.28)	0.530
	T	T	C	0.038	0.045	1.66 (0.44–6.24)	0.450
	T	C	C	0.030	0.008	0.00 (−Inf–Inf)	1.000
	T	C	T	0.026	0.000	0.22 (0.00–1.71 × 10^3^)	0.740
**Urogenital infections**	C	T	-	0.629	0.568	1.00	---
	C	C	-	0.199	0.246	1.35 (0.90–2.02)	0.150
	T	T	-	0.118	0.187	1.70 (1.05–2.75)	**0.031**
	T	C	-	0.054	0.000	0.00 (−Inf–Inf)	1.000
	C	T	T	0.356	0.299	1.00	---
	C	T	C	0.275	0.276	1.30 (0.71–2.39)	0.390
	C	C	T	0.134	0.150	1.41 (0.63–3.14)	0.410
	T	T	T	0.079	0.134	1.91 (0.91–4.00)	0.089
	C	C	C	0.064	0.089	1.57 (0.62–3.99)	0.350
	T	T	C	0.038	0.045	1.58 (0.21–12.03)	0.660
	T	C	C	0.030	0.008	0.23 (0.00–1.33 × 10^3^)	0.740
	T	C	T	0.026	0.000	0.14 (0.00–4.16 × 10^5^)	0.790

^a^ SNP, single nucleotide polymorphism; ^b^ pPROM, preterm prelabor rupture of membranes; ^c^ OR, odds ratio; ^d^ 95% CI, confidence interval; ^e^ *p*-value, statistically significant results are marked in bold; ^f^ Inf, infinity.

## Data Availability

All data and materials, as well as software application, support the published claims and comply with field standards.

## Supplementary Materials

The following are available online at https://www.mdpi.com/article/10.3390/genes12111725/s1, Table S1: Characteristics of women with prelabor rupture of membranes, Table S2: PCR-RFLP assays and genotype profiles for CSF2, FLT1, TLR9, and TFPI polymorphisms, Table S3a: Distribution of genotypes of CSF2, FLT1, TFPI, and TLR9 polymorphisms between women with PROM and healthy controls, Table S3b: Relationship between genotypes of CSF2, FLT1, TFPI, and TLR9 SNPs and the incidence of preterm prelabor rupture of membranes, Table S4: Distribution of genotypes of CSF2, FLT1, TFPI, and TLR9 SNPs between women with term and preterm PROM, Table S5: Distribution of alleles from CSF2, FLT1, TFPI, and TLR9 polymorphisms in women with PROM and healthy controls, Table S6: Incidence of alleles for CSF2, FLT1, TFPI, and TLR9 polymorphisms in women with term and preterm PROM, Table S7: Double-SNP variants for CSF2 and FLT1 polymorphisms and the incidence of PROM, adjusted to the pregnancy disorders, Table S8: Multiple-SNP variants for CSF2, FLT1, TLR9, and TFPI polymorphisms and the incidence of preterm PROM, after adjusting for APTT and PLT parameters, Table S9: Differences in the prevalence of multiple-SNP variants for CSF2, FLT1, TLR9, and TFPI polymorphisms between women with term and preterm PROM, corrected for APTT and PLT parameters, Table S10: Distribution of multiple-SNP variants for CSF2, FLT1, TLR9, and TFPI polymorphisms between women with term and preterm PROM, corrected for pregnancy disorders.

## References

[1] (2020). Prelabor Rupture of Membranes: ACOG Practice Bulletin, Number 217. Obstet Gynecol..

[2] Ghafoor S. (2021). Current and Emerging Strategies for Prediction and Diagnosis of Prelabour Rupture of the Membranes: A Narra-tive Review. Malays. J. Med. Sci..

[3] Meloni A., Palmas F., Barberini L., Mereu R., Deiana S.F., Fais M.F., Noto A., Fattuoni C., Mussap M., Ragusa A. (2018). PROM and Labour Effects on Urinary Metabolome: A Pilot Study. Dis. Markers.

[4] Ocviyanti D., Wahono W.T. (2018). Risk Factors for Neonatal Sepsis in Pregnant Women with Premature Rupture of the Membrane. J. Pregnancy.

[5] Ananth C.V., Joseph K., Oyelese Y., Demissie K., Vintzileos A.M. (2005). Trends in Preterm Birth and Perinatal Mortality Among Singletons: United States, 1989 Through 2000. Obstet. Gynecol..

[6] Choi E.K., Kim S.Y., Heo J.M., Park K.H., Kim H.Y., Choi B.M., Kim H.J. (2021). Perinatal Outcomes Associated with Latency in Late Pre-term Premature Rupture of Membranes. Int. J. Environ. Res. Public Health.

[7] Menon R., Richardson L. (2017). Preterm prelabor rupture of the membranes: A disease of the fetal membranes. Semin. Perinatol..

[8] Günay T., Erdem G., Bilir R.A., Hocaoglu M., Ozdamar O., Turgut A. (2020). The association of the amniotic fluid index (AFI) with perinatal fetal and maternal outcomes in pregnancies complicated by preterm premature rupture of membranes (PPROM). Ginekol. Pol..

[9] Tchirikov M., Schlabritz-Loutsevitch N., Maher J., Buchmann J., Naberezhnev Y., Winarno A.S., Seliger G. (2018). Mid-trimester preterm premature rupture of membranes (PPROM): Etiology, diagnosis, classification, international recommendations of treatment options and outcome. J. Périnat. Med..

[10] Waters T.P., Mercer B.M. (2009). The management of preterm premature rupture of the membranes near the limit of fetal viability. Am. J. Obstet. Gynecol..

[11] Schmitz T., Sentilhes L., Lorthe E., Gallot D., Madar H., Doret-Dion M., Beucher G., Charlier C., Cazanave C., Delorme P. (2019). Preterm premature rupture of the membranes: Guidelines for clinical practice from the French College of Gynaecologists and Obstetricians (CNGOF). Eur. J. Obstet. Gynecol. Reprod. Biol..

[12] Andrys C., Kacerovsky M., Drahosova M., Musilova I., Pliskova L., Hornychova H., Prochazka M., Jacobsson B. (2013). Amniotic fluid soluble Toll-like receptor 2 in pregnancies complicated by preterm prelabor rupture of membranes. J. Matern. Fetal Neonatal Med..

[13] Erez O., Espinoza J., Chaiworapongsa T., Gotsch F., Kusanovic J.P., Than N.G., Mazaki-Tovi S., Vaisbuch E., Papp Z., Yoon B.H. (2008). A link between a hemostatic disorder and preterm PROM: A role for tissue factor and tissue factor pathway inhibitor. J. Matern. Fetal Neonatal Med..

[14] Grote K., Petri M., Liu C., Jehn P., Spalthoff S., Kokemüller H., Luchtefeld M., Tschernig T., Krettek C., Haasper C. (2013). Toll-like receptor 2/6-dependent stimulation of mesen-chymal stem cells promotes angiogenesis by paracrine factors. Eur. Cell Mater..

[15] Kumar D., Moore R.M., Nash A., Springel E., Mercer B.M., Philipson E., Mansour J.M., Moore J.J. (2014). Decidual GM-CSF is a critical common interme-diate necessary for thrombin and TNF induced in-vitro fetal membrane weakening. Placenta.

[16] Kumar D., Schatz F., Moore R.M., Mercer B.M., Rangaswamy N., Mansour J.M., Lockwood C.J., Moore J.J. (2011). The effects of thrombin and cytokines upon the biomechanics and remodeling of isolated amnion membrane, in vitro. Placenta.

[17] Moore R.M., Schatz F., Kumar D., Mercer B.M., Abdelrahim A., Rangaswamy N., Bartel C., Mansour J.M., Lockwood C.J., Moore J.J. (2010). α-lipoic acid inhibits throm-bin-induced fetal membrane weakening in vitro. Placenta.

[18] Fang Q., Liu X., Al-Mugotir M., Kobayashi T., Abe S., Kohyama T. (2006). Thrombin and TNF-α/IL-1beta synergistically in-duce fibroblast-mediated collagen gel degradation. Am. J. Respir. Cell Mol. Biol..

[19] Galis Z.S., Kranzhöfer R., Fenton J.W., Libby P. (1997). Thrombin Promotes Activation of Matrix Metalloproteinase-2 Produced by Cultured Vascular Smooth Muscle Cells. Arter. Thromb. Vasc. Biol..

[20] Han C.S., Schatz F., Lockwood C.J. (2011). Abruption-Associated Prematurity. Clin. Perinatol..

[21] Mackenzie A.P., Schatz F., Krikun G., Funai E.F., Kadner S., Lockwood C.J. (2004). Mechanisms of abruption-induced premature rupture of the fetal membranes: Thrombin enhanced decidual matrix metalloproteinase-3 (stromelysin-1) expression. Am. J. Obstet. Gynecol..

[22] Stephenson C.D., Lockwood C.J., Ma Y., Guller S. (2005). Thrombin-dependent regulation of matrix metalloproteinase (MMP)-9 levels in human fetal membranes. J. Matern. Fetal Neonatal Med..

[23] Erez O., Romero R., Vaisbuch E., Kusanovic J.P., Mazaki-Tovi S., Chaiworapongsa T., Gotsch F., Fareed J., Hoppensteadt D., Than N.G. (2010). High tissue factor activity and low tissue factor pathway inhibitor concentrations in patients with preterm labor. J. Matern. Fetal Neonatal Med..

[24] Gomez-Lopez N., Hernandez-Santiago S., Lobb A.P., Olson D.M., Vadillo-Ortega F. (2012). Normal and Premature Rupture of Fetal Membranes at Term Delivery Differ in Regional Chemotactic Activity and Related Chemokine/Cytokine Production. Reprod. Sci..

[25] Musilova I., Pliskova L., Kutova R., Hornychova H., Jacobsson B., Kacerovsky M. (2016). Ureaplasma species and Mycoplasma hominis in cervical fluid of pregnancies complicated by preterm prelabor rupture of membranes. J. Mater. Fetal Neonatal Med..

[26] Oh K.J., Romero R., Park J.Y., Hong J.S., Yoon B.H. (2019). The earlier the gestational age, the greater the intensity of the in-tra-amniotic inflammatory response in women with preterm premature rupture of membranes and amniotic fluid infection by Ureaplasma species. J. Perinat. Med..

[27] Perni S.C., Vardhana S., Korneeva I., Tuttle S.L., Paraskevas L.-R., Chasen S.T., Kalish R.B., Witkin S.S. (2004). Mycoplasma hominis and Ureaplasma urealyticum in midtrimester amniotic fluid: Association with amniotic fluid cytokine levels and pregnancy outcome. Am. J. Obstet. Gynecol..

[28] Tantengco O.A.G., Yanagihara I. (2019). Current understanding and treatment of intra-amniotic infection with Ureaplasma spp. J. Obstet. Gynaecol. Res..

[29] Fitzgerald K.A., Kagan J.C. (2020). Toll-like Receptors and the Control of Immunity. Cell.

[30] Mukherjee S., Huda S., Sinha Babu S.P. (2019). Toll-like receptor polymorphism in host immune response to infectious diseases: A review. Scand. J. Immunol..

[31] Vijay K. (2018). Toll-like receptors in immunity and inflammatory diseases: Past, present, and future. Int. Immunopharmacol..

[32] Kacerovsky M., Andrys C., Hornychová H., Pliskova L., Lancz K., Musilová I.K., Drahosova M., Bolehovska R., Tambor V., Jacobsson B. (2012). Amniotic fluid soluble Toll-like receptor 4 in pregnancies complicated by preterm prelabor rupture of the membranes. J. Matern. Fetal Neonatal Med..

[33] He B., Yang X., Li Y., Huang D., Xu X., Yang W., Dai Y., Zhang H., Chen Z., Cheng W. (2018). TLR9 (Toll-Like Receptor 9) Agonist Suppresses Angiogenesis by Dif-ferentially Regulating VEGFA (Vascular Endothelial Growth Factor A) and sFLT1 (Soluble Vascular Endothelial Growth Fac-tor Receptor 1) in Preeclampsia. Hypertension.

[34] Mohamed F.E.-Z.A., Hammad S., Luong T.V., Dewidar B., Al-Jehani R., Davies N., Dooley S., Jalan R. (2020). Expression of TLR-2 in hepatocellular carcinoma is associated with tumour proliferation, angiogenesis and Caspase-3 expression. Pathol. Res. Pract..

[35] Zhao L., Ma R., Zhang L., Yuan X., Wu J., He L., Liu G., Du R. (2019). Inhibition of HIF-1a-mediated TLR4 activation decreases apoptosis and promotes angiogenesis of placental microvascular endothelial cells during severe pre-eclampsia pathogenesis. Placenta.

[36] Saber T., Veale U.J., Balogh E., McCormick J., NicAnUltaigh S., Connolly M., Fearon U. (2011). Toll-Like Receptor 2 Induced Angiogenesis and Invasion Is Mediated through the Tie_2_ Signalling Pathway in Rheumatoid Arthritis. PLoS ONE.

[37] Grote K., Schuett H., Salguero G., Grothusen C., Jagielska J., Drexler H., Mühlradt P.F., Schieffer B. (2010). Toll-like receptor 2/6 stimulation promotes angiogenesis via GM-CSF as a potential strategy for immune defense and tissue regeneration. Blood.

[38] El Kebir D., Damlaj A., Makhezer N., Filep J.G. (2015). Toll-Like Receptor 9 Signaling Regulates Tissue Factor and Tissue Factor Pathway Inhibitor Expression in Human Endothelial Cells and Coagulation in Mice. Crit. Care Med..

[39] Harmon Q.E., Engel S.M., Olshan A.F., Moran T., Stuebe A.M., Luo J., Wu M.C., Avery C.L. (2013). Association of polymorphisms in natural killer cell-related genes with preterm birth. Am. J. Epidemiol..

[40] Gómez L.M., Sammel M.D., Appleby D.H., Elovitz M.A., Baldwin D.A., Jeffcoat M.K., Macones G.A., Parry S. (2010). Evidence of a gene-environment interaction that predisposes to spontaneous preterm birth: A role for asymptomatic bacterial vaginosis and DNA variants in genes that control the inflammatory response. Am. J. Obstet. Gynecol..

[41] Frey H.A., Stout M.J., Pearson L.N., Tuuli M.G., Cahill A.G., Strauss J.F., Gomez L.M., Parry S., Allsworth J.E., Macones G.A. (2016). Genetic variation associated with preterm birth in African-American women. Am. J. Obstet. Gynecol..

[42] Amin-Beidokhti M., Gholami M., Abedin-Do A., Pirjani R., Sadeghi H., Karamoddin F., Yassaee V.R., Mirfakhraie R. (2018). An intron variant in the FLT1 gene increases the risk of preeclampsia in Iranian women. Clin. Exp. Hypertens..

[43] Majewska M., Lipka A., Paukszto L., Jastrzebski J.P., Szeszko K., Gowkielewicz M., Lepiarczyk E., Jozwik M., Majewski M.K. (2019). Placenta Transcriptome Profiling in Intrauterine Growth Restriction (IUGR). Int. J. Mol. Sci..

[44] Cao Y., Zhang Z., Xu J., Yuan W., Wang J., Huang X., Shen Y., Du J. (2013). The association of idiopathic recurrent pregnancy loss with pol-ymorphisms in hemostasis-related genes. Gene.

[45] Guerra-Shinohara E.M., Bertinato J.F., Bueno C.T., da Silva K.C., de Carvalho M.H.B., Francisco R.P.V., Zugaib M., Cerda A., Morelli V.M. (2012). Polymorphisms in antithrombin and in tissue factor pathway inhibitor genes are associated with recurrent pregnancy loss. Thromb Haemost..

[46] Karody V.R., Reese S., Kumar N., Liedel J., Jarzembowski J., Sampath V. (2016). A toll-like receptor 9 (rs352140) variant is associated with placental inflammation in newborn infants. J. Matern. Fetal Neonatal Med..

[47] Razdaibiedina A., Khobzey M., Tkachenko V., Vorobiova I. (2018). Effects of Single-Nucleotide Polymorphisms in Cytokine, Toll-Like Receptor, and Progesterone Receptor Genes on Risk of Miscarriage. Obstet. Gynecol. Int..

[48] Mockenhaupt F.P., Hamann L., von Gaertner C., Bedu-Addo G., von Kleinsorgen C., Schumann R.R., Bienzle U. (2006). Common poly-morphisms of toll-like receptors 4 and 9 are associated with the clinical manifestation of malaria during pregnancy. J. Infect. Dis..

[49] Moatti D., Haidar B., Fumeron F., Gauci L., Boudvillain O., Seknadji P., Olliver V., Aumont M.C., De Prost D. (2000). A new T-287C polymorphism in the 5′ regulatory region of the tissue factor pathway inhibitor gene. Association study of the T-287C and C-399T polymorphisms with coronary artery disease and plasma TFPI levels. Thromb. Haemost..

[50] Pandey S., Mittal B., Srivastava M., Singh S., Srivastava K., Lal P., Mittal R.D. (2010). Evaluation of Toll-like receptors 3 (c.1377C/T) and 9 (G2848A) gene polymorphisms in cervical cancer susceptibility. Mol. Biol. Rep..

[51] Saeki H., Tsunemi Y., Asano N., Nakamura K., Sekiya T., Hirai K., Kakinuma T., Fujita H., Kagami S., Tamaki K. (2006). Analysis of GM-CSF gene polymorphisms (3606T/C and 3928C/T) in Japanese patients with atopic dermatitis. Clin. Exp. Dermatol..

[52] SNPStats Software. https://www.snpstats.net/start.htm.

[53] Srinivas S.K., Morrison A., Andrela C.M., Elovitz M. (2010). Allelic variations in angiogenic pathway genes are associated with preeclampsia. Am. J. Obstet. Gynecol..

[54] Amosco M.D., Villar V.A.M., Naniong J.M.A., David-Bustamante L.M.G., Jose P.A., Palmes-Saloma C.P. (2016). VEGF-A and VEGFR1 SNPs associate with preeclampsia in a Philippine population. Clin. Exp. Hypertens..

[55] Opstad T.B., Pettersen A.A., Bratseth V., Arnesen H., Seljeflot I. (2010). The influence of tissue factor and tissue factor pathway in-hibitor polymorphisms on thrombin generation in stable coronary artery disease. Pathophysiol. Haemost. Thromb..

[56] Keren-Politansky A., Breizman T., Brenner B., Sarig G., Drugan A. (2014). The coagulation profile of preterm delivery. Thromb. Res..

[57] Ekin A., Gezer C., Kulhan G., Avcı M.E., Taner C.E. (2014). Can platelet count and mean platelet volume during the first trimester of pregnancy predict preterm premature rupture of membranes?. J. Obstet. Gynaecol. Res..

[58] Gasparyan A.Y., Ayvazyan L., Mikhailidis D.P., Kitas G.D. (2011). Mean platelet volume: A link between thrombosis and inflamma-tion?. Curr. Pharm. Des..

[59] Roszak A., Lianeri M., Sowińska A., Jagodziński P.P. (2012). Involvement of Toll-like Receptor 9 polymorphism in cervical cancer development. Mol. Biol. Rep..

[60] Wu J., Cui H., Dick A.D., Liu L. (2014). TLR9 Agonist Regulates Angiogenesis and Inhibits Corneal Neovascularization. Am. J. Pathol..

[61] Bouvier D., Forest J.-C., Blanchon L., Bujold E., Pereira B., Bernard N., Gallot D., Sapin V., Giguère Y. (2019). Risk Factors and Outcomes of Preterm Premature Rupture of Membranes in a Cohort of 6968 Pregnant Women Prospectively Recruited. J. Clin. Med..

[62] Luisi S., Giorgi M., Riggi S., Messina G., Severi F.M. (2020). Neonatal outcome in pregnancy hypotiroidee women. Gynecol. Endocrinol..

[63] Merello M., Lotte L., Gonfrier S., Trolli S.E.D., Casagrande F., Ruimy R., Bongain A. (2019). Enterobacteria vaginal colonization among patients with preterm premature rupture of membranes from 24 to 34 weeks of gestation and neonatal infection risk. J. Gynecol. Obstet. Hum. Reprod..

[64] Muche A.A., Olayemi O.O., Gete Y.K. (2020). Effects of gestational diabetes mellitus on risk of adverse maternal outcomes: A prospec-tive cohort study in Northwest Ethiopia. BMC Pregnancy Childbirth.

[65] Workineh Y., Birhanu S., Kerie S., Ayalew E., Yihune M. (2018). Determinants of premature rupture of membrane in Southern Ethiopia, 2017: Case control study design. BMC Res. Notes.

[66] El-Achi V., De Vries B., O’Brien C., Park F., Tooher J., Hyett J. (2020). First-Trimester Prediction of Preterm Prelabour Rupture of Membranes. Fetal Diagn. Ther..

[67] Zhang X., Liao Q., Wang F., Li D. (2018). Association of gestational diabetes mellitus and abnormal vaginal flora with adverse pregnancy outcomes. Medicine.

[68] Getahun D., Ananth C.V., Oyelese Y., Peltier M.R., Smulian J.C., Vintzileos A.M. (2007). Acute and chronic respiratory diseases in pregnancy: Associations with spontaneous premature rupture of membranes. J. Matern. Fetal Neonatal Med..

[69] Hnat M.D., Mercer B.M., Thurnau G., Goldenberg R., Thom E.A., Meis P.J., Moawad A.H., Iams J.D., Van Dorsten J.P. (2005). Perinatal outcomes in women with preterm rupture of membranes between 24 and 32 weeks of gestation and a history of vaginal bleeding. Am. J. Obstet. Gynecol..

[70] Liu L., Wang L., Yang W., Ni W., Jin L., Liu J., Ren A. (2019). Gestational hypertension and pre-eclampsia and risk of spontaneous premature rupture of membranes: A population-based cohort study. Int. J. Gynecol. Obstet..

[71] Ahmed S.R., El-Sammani M., Al-Sheeha M.A., Aitallah A.S., Jabin K.F., Ahmed S.R. (2012). Pregnancy outcome in women with threatened miscarriage: A year study. Mater Sociomed..

[72] Evrenos A.N., Gungor A.N.C., Gulerman C., Cosar E. (2013). Obstetric outcomes of patients with abortus imminens in the first trimester. Arch. Gynecol. Obstet..

[73] Brown R.G., Marchesi J.R., Lee Y.S., Smith A., Lehne B., Kindinger L.M., Terzidou V., Holmes E., Nicholson J.K., Bennett P.R. (2018). Vaginal dysbiosis increases risk of preterm fetal membrane rupture, neonatal sepsis and is exacerbated by erythromycin. BMC Med..

[74] Brown R.G., Al-Memar M., Marchesi J.R., Lee Y.S., Smith A., Chan D., Lewis H., Kindinger L., Terzidou V., Bourne T. (2019). Establishment of vaginal microbiota composition in early pregnancy and its association with subsequent preterm prelabor rupture of the fetal membranes. Transl. Res..

