# Supplementary material for: Single Nucleotide Polymorphisms from CSF2, FLT1, TFPI and TLR9 Genes Are Associated with Prelabor Rupture of Membranes
Source: Genes (Basel). 2021 Oct 28;12(11):1725. doi: 10.3390/genes12111725 (PMC8620696; doi:10.3390/genes12111725)
Supplement: Supplementary file 1 [file genes-12-01725-s001.zip › genes-1415956-supplementary.pdf]

Table S1. Characteristics of women with prelabor rupture of membranes.

		PROM[a] cases	tPROM[b]	pPROM[c]	*P*-value[d]
**Number**		180	54	126	
**Age [years]**		29.83 ± 5.50	27.48 ± 4.58	30.83 ± 5.57	**≤ 0.001**
**No.[e] of pregnancy, n (%)**	**1**	87 (48.3%)	40 (74.1%)	47 (37.3%)	**≤ 0.001**
	**2**	55 (30.6%)	10 (18.5%)	45 (35.7%)	
	**3**	26 (14.4%)	4 (7.4%)	22 (17.5%)	
	**4**	7 (3.9%)	0 (0.0%)	7 (5.5%)	
	**5**	3 (1.7%)	0 (0.0%)	3 (2.4%)	
	**6**	2 (1.1%)	0 (0.0%)	2 (1.6%)	
**Pregnancy disorders, n (%)**	**Asthma and respiratory system infections**	10 (5.6%)	4 (7.4%)	6 (4.8%)	0.348
	**Bleeding**	7 (3.9%)	2 (3.7%)	5 (4.0%)	0.648
	**Diabetes mellitus**	18 (10.0%)	2 (3.7%)	16 (12.7)	0.051
	**Hypertension**	19 (10.6%)	6 (11.1%)	13 (10.3%)	0.874
	**Hypothyroidism**	36 (20.0%)	11 (20.4%)	25 (19.8%)	0.935
	**Serological conflict**	4 (2.2%)	2 (3.7%)	2 (1.6%)	0.348
	**Threatened miscarriage**	15 (8.3)	3 (5.6%)	12 (9.5%)	0.287
	**Urogenital infections**	20 (11.1%)	9 (16.7%)	11 (8.7%)	0.121
**APTT [s][f]**		27.7 (21.7-44.5)	27.5 (22.5-33.7)	28.1 (21.7-44.5)	0.372
**Platelet parameters**	**No. [x10^9/L]**	214 (59-457)	191 (89-314)	220.5 (59-457)	**0.028**
	**PDW [fL][g]**	13.3 (8.8-24.9)	15.9 (10.7-21.8)	12.9 (8.8-24.9)	**≤ 0.001**
	**MPV [fL][h]**	11.1 (8.8-14.6)	12.0 (9.7-13.7)	10.9 (8.8-14.6)	**≤ 0.001**
	**PCT [%][i]**	0.23 (0.06-0.50)	0.22 (0.12-0.32)	0.23 (0.06-0.50)	0.503
**Delivery, n (%)**	**Weeks of pregnancy**	35 (17-41)	39 (37-41)	33 (17-40)	**≤ 0.001**
	**Natural**	78 (44.8%)	29 (53.7%)	49 (40.8%)	0.114
	**C-section[j]**	96 (55.2)	25 (46.3%)	71 (59.2%)	
**Fetal sex, n (%)**	**Female**	72 (42.6%)	22 (40.7%)	50 (43.5%)	0.737
	**Male**	97 (57.4%)	32 (59.3%)	65 (56.5%)	
**Neonatal data**	**Weight [percentiles]**	63.5 (0-100)	69.5 (1-100)	56.5 (0-100)	0.240
	**Apgar in 1 min**	9 (0-10)	10 (2-10)	7 (0-10)	**≤ 0.001**
	**Apgar in 5 min**	9 (0-10)	10 (3-10)	8 (0-10)	**≤ 0.001**

[a.] PROM, prelabor rupture of membranes; [b.] tPROM, term PROM; [c.] pPROM, preterm PROM; [d.] *P*-value, statistically significant results are marked in bold; [e.] No., number; [f.] APTT [s], activated partial thromboplastin time [second]; [g.] PDW, platelet distribution width; [h.] MPV, mean platelet volume; [i.] PCT, plateletcrit; [j.] C-section, caesarean section.

Table S2. PCR-RFLP assays and genotype profiles for *CSF2*, *FLT1*, *TLR9*, and *TFPI* polymorphisms.

Gene	SNP[a]	Alleles	MAF[b] [%]	Primer sequences (5'-3')	Annealing temperature [ºC]	Restriction enzyme	Genotypes [bp][c]	Agarose gel [%]
CSF2	**rs25881**	C>T	17.9	F: TTCTGGCAGGACTTTCCTCT<br>R: AGTGCATAGCTCTGCCAGTG	58	BlpI	CC: 292<br>CT: 292, 170, 122<br>TT: 170, 122	2.5
FLT1	**rs722503**	C>T	26.8	F: TCCGCCTGCATTTTGAACAACTAAGTAG<br>R: GGTCTCCTTGGTATTCAAGCACACGTAA	58	AvaII	CC: 199, 169<br>CT: 368, 199, 169<br>TT: 368	2.5
TLR9	**rs352140**	C>T	48.0	F: AAGCTGGACCTCTACCACGA<br>R: TTGGCTGTGGATGTTGTT	58	BstUI	CC: 135, 42<br>CT: 177, 135, 42<br>TT: 177	3.0
TFPI	**C-399T**	C>T	-	F: ACACCTGCAATAGATAATGCATTACAGAGT<br>R: TGAATAGCTATTTCCTTTACTTTCC	55	HinfI	CC: 235, 27<br>CT: 262, 235, 27<br>TT: 262	3.0

[a.] SNP, single nucleotide polymorphism; [b.] MAF, minor allele frequency; [c.] bp, base pair.

Table S3a. Distribution of genotypes of *CSF2*, *FLT1*, *TFPI*, and *TLR9* polymorphisms between women with PROM and healthy controls.

Polymorphism	Genetic model	Genotype	Genotype prevalence, n (%)[a]		OR[b] (95 % CI[c])	*P*-value[d]
			Controls	Cases		
CSF2 **rs25881**	Codominant	CC	127 (70.6%)	122 (67.8%)	1.00	0.650
		CT	44 (24.4%)	51 (28.3%)	1.21 (0.75-1.94)	
		TT	9 (5.0%)	7 (3.9%)	0.81 (0.29-2.24)	
	Dominant	CC	127 (70.6%)	122 (67.8%)	1.00	0.570
		CT-TT	53 (29.4%)	58 (32.2%)	1.14 (0.73-1.78)	
	Recessive	CC-CT	171 (95.0%)	173 (96.1%)	1.00	0.610
		TT	9 (5.0%)	7 (3.9%)	0.77 (0.28-2.11)	
	Overdominant	CC-TT	136 (75.6%)	129 (71.7%)	1.00	0.400
		CT	44 (24.4%)	51 (28.3%)	1.22 (0.76-1.95)	
FLT1 **rs722503**	Codominant	TT	104 (57.8%)	107 (59.4%)	1.00	0.440
		CT	61 (33.9%)	64 (35.6%)	1.02 (0.66-1.59)	
		CC	15 (8.3%)	9 (5.0%)	0.58 (0.24-1.39)	
	Dominant	TT	104 (57.8%)	107 (59.4%)	1.00	0.750
		CT-CC	76 (42.2%)	73 (40.6%)	0.93 (0.61-1.42)	
	Recessive	TT-CT	165 (91.7%)	171 (95.0%)	1.00	0.200
		CC	15 (8.3%)	9 (5.0%)	0.58 (0.25-1.36)	
	Overdominant	TT-CC	119 (66.1%)	116 (64.4%)	1.00	0.740
		CT	61 (33.9%)	64 (35.6%)	1.08 (0.70-1.66)	
TFPI **C-399T**	Codominant	CC	151 (83.9%)	146 (81.1%)	1.00	0.420
		CT	29 (16.1%)	33 (18.3%)	1.18 (0.68-2.04)	
		TT	0 (0.0%)	1 (0.6%)	NA[e] (0.00-NA)	
	Dominant	CC	151 (83.9%)	146 (81.1%)	1.00	0.490
		CT-TT	29 (16.1%)	34 (18.9%)	1.21 (0.70-2.09)	

	Recessive	CC-CT	180 (100.0%)	179 (99.4%)	1.00	0.240
		TT	0 (0.0%)	1 (0.6%)	NA (0.00-NA)	
	Overdominant	CC-TT	151 (83.9%)	147 (81.7%)	1.00	0.580
		CT	29 (16.1%)	33 (18.3%)	1.17 (0.68-2.02)	
TLR9 **rs352140**	Codominant	TT	57 (31.7%)	46 (25.6%)	1.00	0.360
		CT	100 (55.6%)	113 (62.8%)	1.40 (0.87-2.25)	
		CC	23 (12.8%)	21 (11.7%)	1.13 (0.56-2.30)	
	Dominant	TT	57 (31.7%)	46 (25.6%)	1.00	0.200
		CT-CC	123 (68.3%)	134 (74.4%)	1.35 (0.85-2.14)	
	Recessive	TT-CT	157 (87.2%)	159 (88.3%)	1.00	0.750
		CC	23 (12.8%)	21 (11.7%)	0.90 (0.48-1.70)	
	Overdominant	TT-CC	80 (44.4%)	67 (37.2%)	1.00	0.160
		CT	100 (55.6%)	113 (62.8%)	1.35 (0.89-2.06)	

[a.] n, number; [b.] OR, odds ratio; [c.] 95% CI, confidence interval; [d.] *P*-value, $P \leq 0.050$ is considered significant; [e.] NA, not analyzed.

Table S3b. Relationship between genotypes of *CSF2*, *FLT1*, *TFPI*, and *TLR9* SNPs and the incidence of preterm prelabor rupture of membranes.

Polymorphism	Genetic model	Genotype	Genotype prevalence, n (%)[a]		OR[c] (95 % CI[d])	*P*-value[e]
			Controls	pPROM[b] cases		
CSF2 **rs25881**	Codominant	CC	127 (70.6%)	82 (65.1%)	1.00	0.180
		CT	44 (24.4%)	41 (32.5%)	1.44 (0.87-2.40)	
		TT	9 (5.0%)	3 (2.4%)	0.52 (0.14-1.96)	
	Dominant	CC	127 (70.6%)	82 (65.1%)	1.00	0.310
		CT-TT	53 (29.4%)	44 (34.9%)	1.29 (0.79-2.09)	
	Recessive	CC-CT	171 (95.0%)	123 (97.6%)	1.00	0.230
		TT	9 (5.0%)	3 (2.4%)	0.46 (0.12-1.75)	
	Overdominant	CC-TT	136 (75.6%)	85 (67.5%)	1.00	0.120
		CT	44 (24.4%)	41 (32.5%)	1.49 (0.90-2.47)	
FLT1 **rs722503**	Codominant	TT	104 (57.8%)	71 (56.4%)	1.00	0.550
		CT	61 (33.9%)	48 (38.1%)	1.15 (0.71-1.87)	
		CC	15 (8.3%)	7 (5.6%)	0.68 (0.27-1.76)	
	Dominant	TT	104 (57.8%)	71 (56.4%)	1.00	0.800
		CT-CC	76 (42.2%)	55 (43.6%)	1.06 (0.67-1.68)	
	Recessive	TT-CT	165 (91.7%)	119 (94.4%)	1.00	0.350
		CC	15 (8.3%)	7 (5.6%)	0.65 (0.26-1.64)	
	Overdominant	TT-CC	119 (66.1%)	78 (61.9%)	1.00	0.450
		CT	61 (33.9%)	48 (38.1%)	1.20 (0.75-1.93)	
TFPI **C-399T**	Codominant	CC	151 (83.9%)	103 (81.8%)	1.00	0.390
		CT	29 (16.1%)	22 (17.5%)	1.11 (0.61-2.04)	
		TT	0 (0.0%)	1 (0.8%)	NA[f] (0.00-NA)	
	Dominant	CC	151 (83.9%)	103 (81.8%)	1.00	0.620
		CT-TT	29 (16.1%)	23 (18.2%)	1.16 (0.64-2.12)	
	Recessive	CC-CT	180 (100.0%)	125 (99.2%)	1.00	0.180

		TT	0 (0.0%)	1 (0.8%)	NA (0.00-NA)	
	Overdominant	CC-TT	151 (83.9%)	104 (82.5%)	1.00	0.760
		CT	29 (16.1%)	22 (17.5%)	1.10 (0.60-2.02)	
TLR9 **rs352140**	Codominant	TT	57 (31.7%)	33 (26.2%)	1.00	0.300
		CT	100 (55.6%)	81 (64.3%)	1.40 (0.83-2.35)	
		CC	23 (12.8%)	12 (9.5%)	0.90 (0.40-2.04)	
	Dominant	TT	57 (31.7%)	33 (26.2%)	1.00	0.300
		CT-CC	123 (68.3%)	93 (73.8%)	1.31 (0.79-2.17)	
	Recessive	TT-CT	157 (87.2%)	114 (90.5%)	1.00	0.370
		CC	23 (12.8%)	12 (9.5%)	0.72 (0.34-1.50)	
	Overdominant	TT-CC	80 (44.4%)	45 (35.7%)	1.00	0.130
		CT	100 (55.6%)	81 (64.3%)	1.44 (0.90-2.30)	

[a.] n, number; [b.] pPROM, preterm prelabor rupture of membranes; [c.] OR, odds ratio; [d.] 95% CI, confidence interval; [e.] *P*-value, $P \leq 0.050$ is considered significant; [f.] NA, not analyzed.

Table S4. Distribution of genotypes of *CSF2*, *FLT1*, *TFPI*, and *TLR9* SNPs between women with term and preterm PROM.

Polymorphism	Genetic model	Genotype	Genotype prevalence, n (%)[a]		OR[d] (95 % CI[e])	*P*-value[f]
			tPROM[b]	pPROM[c]		
***CSF2* rs25881**	Codominant	CC	40 (74.1%)	82 (65.1%)	1.00	0.064
		CT	10 (18.5%)	41 (32.5%)	2.00 (0.91-4.40)	
		TT	4 (7.4%)	3 (2.4%)	0.37 (0.08-1.71)	
	Dominant	CC	40 (74.1%)	82 (65.1%)	1.00	0.230
		CT-TT	14 (25.9%)	44 (34.9%)	1.53 (0.75-3.12)	
	Recessive	CC-CT	50 (92.6%)	123 (97.6%)	1.00	0.130
		TT	4 (7.4%)	3 (2.4%)	0.30 (0.07-1.41)	
	Overdominant	CC-TT	44 (81.5%)	85 (67.5%)	1.00	0.050
		CT	10 (18.5%)	41 (32.5%)	2.12 (0.97-4.64)	
***FLT1* rs722503**	Codominant	TT	36 (66.7%)	71 (56.4%)	1.00	0.420
		CT	16 (29.6%)	48 (38.1%)	1.52 (0.76-3.04)	
		CC	2 (3.7%)	7 (5.6%)	1.77 (0.35-8.98)	
	Dominant	TT	36 (66.7%)	71 (56.4%)	1.00	0.190
		CT-CC	18 (33.3%)	55 (43.6%)	1.55 (0.80-3.02)	
	Recessive	TT-CT	52 (96.3%)	119 (94.4%)	1.00	0.590
		CC	2 (3.7%)	7 (5.6%)	1.53 (0.31-7.61)	
	Overdominant	TT-CC	38 (70.4%)	78 (61.9%)	1.00	0.270
		CT	16 (29.6%)	48 (38.1%)	1.46 (0.74-2.90)	
***TFPI* C-399T**	Codominant	CC	43 (79.6%)	103 (81.8%)	1.00	0.640
		CT	11 (20.4%)	22 (17.5%)	0.83 (0.37-1.87)	
		TT	0 (0.0%)	1 (0.8%)	NA[g] (0.00-NA)	
	Dominant	CC	43 (79.6%)	103 (81.8%)	1.00	0.740
		CT-TT	11 (20.4%)	23 (18.2%)	0.87 (0.39-1.95)	

	Recessive	CC-CT	54 (100.0%)	125 (99.2%)	1.00	0.400
		TT	0 (0.0%)	1 (0.8%)	NA (0.00-NA)	
	Overdominant	CC-TT	43 (79.6%)	104 (82.5%)	1.00	0.650
		CT	11 (20.4%)	22 (17.5%)	0.83 (0.37-1.85)	
***TLR9* rs352140**	Codominant	TT	13 (24.1%)	33 (26.2%)	1.00	0.410
		CT	32 (59.3%)	81 (64.3%)	1.00 (0.47-2.13)	
		CC	9 (16.7%)	12 (9.5%)	0.53 (0.18-1.54)	
	Dominant	TT	13 (24.1%)	33 (26.2%)	1.00	0.760
		CT-CC	41 (75.9%)	93 (73.8%)	0.89 (0.43-1.87)	
	Recessive	TT-CT	45 (83.3%)	114 (90.5%)	1.00	0.180
		CC	9 (16.7%)	12 (9.5%)	0.53 (0.21-1.33)	
	Overdominant	TT-CC	22 (40.7%)	45 (35.7%)	1.00	0.520
		CT	32 (59.3%)	81 (64.3%)	1.24 (0.64-2.38)	

[a.] n, number; [b.] tPROM, term prelabor rupture of membranes; [c.] pPROM, preterm PROM; [d.] OR, odds ratio; [e.] 95% CI, confidence interval; [f.] *P*-value, $P \leq 0.050$ is considered significant; [g.] NA, not analyzed.

Table S5. Distribution of alleles from *CSF2*, *FLT1*, *TFPI*, and *TLR9* polymorphisms in women with PROM and healthy controls.

Polymorphism	Allele	No.[a] of alleles (%)		Chi-Square	*P*-value[c]	No. of alleles (%)	Chi-Square	*P*-value
		Controls	PROM[b] cases			pPROM[d] cases		
CSF2 **rs25881**	C<br>T	298 (82.8)<br>62 (17.2)	295 (81.9)<br>65 (18.1)	0.086	0.769	205 (81.3)<br>47 (18.7)	0.207	0.649
FLT1 **rs722503**	C<br>T	91 (25.3)<br>269 (74.7)	82 (22.8)<br>278 (77.2)	0.616	0.432	62 (24.6)<br>190 (75.4)	0.036	0.850
TFPI **C-399T**	C<br>T	331 (91.9)<br>29 (8.1)	325 (90.2)<br>35 (9.8)	0.617	0.432	228 (90.5)<br>24 (9.5)	0.404	0.525
TLR9 **rs352140**	C<br>T	146 (40.6)<br>214 (59.4)	155 (43.1)<br>205 (56.9)	0.462	0.496	105 (41.7)<br>147 (58.3)	0.076	0.783

[a.] No., number; [b.] PROM, prelabor rupture of membranes; [c.] *P*-value, $P \leq 0.050$ is considered significant; [d.] pPROM, preterm PROM.

Table S6. Incidence of alleles for *CSF2*, *FLT1*, *TFPI*, and *TLR9* polymorphisms in women with term and preterm PROM.

Polymorphism	Allele	No.[a] of alleles (%)		Chi-Square	*P*-value[d]
		tPROM[b]	pPROM[c]		
CSF2	C	90 (83.3)	205 (81.3)	0.086	0.769
**rs25881**	T	18 (16.7)	47 (18.7)		
FLT1	C	20 (18.5)	62 (24.6)	0.616	0.432
**rs722503**	T	88 (81.5)	190 (75.4)		
TFPI	C	97 (89.8)	228 (90.5)	0.617	0.432
**C-399T**	T	11 (10.2)	24 (9.5)		
TLR9	C	50 (46.3)	105 (41.7)	0.462	0.496
**rs352140**	T	58 (53.7)	147 (58.3)		

[a.] No., number; [b.] tPROM, term prelabor rupture of membranes; [c.] pPROM, preterm PROM; [d.] *P*-value, $P \leq 0.050$ is considered significant.

Table S7. Double-SNP variants for *CSF2* and *FLT1* polymorphisms and the incidence of PROM, adjusted to the pregnancy disorders.

Pregnancy disorders	Polymorphisms / Alleles		Multiple-SNP[a] variant frequency		OR[c] (95 % CI[d])	*P*-value[e]
	***CSF2* rs25881**	***FLT1* rs722503**	**Controls**	**PROM[b] cases**		
**Asthma and respiratory system infections**	C	T	0.629	0.592	1.00	---
	C	C	0.199	0.228	1.23 (0.85 - 1.79)	0.280
	T	T	0.118	0.181	1.60 (1.04 - 2.48)	**0.034**
	T	C	0.054	0.000	0.00 (-Inf[f] - Inf)	1.000
**Bleeding**	C	T	0.629	0.592	1.00	---
	C	C	0.199	0.228	1.21 (0.83 - 1.76)	0.310
	T	T	0.118	0.181	1.59 (1.03 - 2.45)	**0.037**
	T	C	0.054	0.000	0.00 (-Inf - Inf)	1.000
**Diabetes mellitus**	C	T	0.629	0.592	1.00	---
	C	C	0.199	0.228	1.21 (0.83 - 1.76)	0.320
	T	T	0.118	0.181	1.58 (1.03 - 2.44)	**0.038**
	T	C	0.054	0.000	0.00 (-Inf - Inf)	1.000
**Hypertension**	C	T	0.629	0.592	1.00	---
	C	C	0.199	0.228	1.21 (0.83 - 1.75)	0.320
	T	T	0.118	0.181	1.57 (1.02 - 2.42)	**0.041**
	T	C	0.054	0.000	0.00 (-Inf - Inf)	1.000
**Hypothyroidism**	C	T	0.629	0.592	1.00	---
	C	C	0.199	0.228	1.21 (0.83 - 1.76)	0.320
	T	T	0.118	0.181	1.58 (1.02 - 2.44)	**0.040**

	T	C	0.054	0.000	0.00 (-Inf - Inf)	1.000
**Serological conflict**	C	T	0.629	0.592	1.00	---
	C	C	0.199	0.228	1.19 (0.82 - 1.74)	0.360
	T	T	0.118	0.181	1.55 (1.00 - 2.40)	**0.049**
	T	C	0.054	0.000	0.00 (-Inf - Inf)	1.000
**Threatened miscarriage**	C	T	0.629	0.592	1.00	---
	C	C	0.199	0.228	1.21 (0.83 - 1.76)	0.330
	T	T	0.118	0.181	1.60 (1.03 - 2.48)	**0.039**
	T	C	0.054	0.000	0.00 (-Inf - Inf)	1.000
**Urogenital infections**	C	T	0.629	0.592	1.00	---
	C	C	0.199	0.228	1.21 (0.83 - 1.76)	0.310
	T	T	0.118	0.181	1.58 (1.02 - 2.44)	**0.039**
	T	C	0.054	0.000	0.00 (-Inf - Inf)	1.000

[a.] SNP, single nucleotide polymorphism; [b.] PROM, prelabor rupture of membranes; [c.] OR, odds ratio; [d.] 95% CI, confidence interval; [e.] *P*-value, statistically significant results are marked in bold; [f.] Inf, infinity.

Table S8. Multiple-SNP variants for *CSF2*, *FLT1*, *TLR9*, and *TFPI* polymorphisms and the incidence of preterm PROM, after adjusting for APTT and PLT parameters.

Categorical covariate	Polymorphisms / Alleles				Multiple-SNP[a] variant frequency		OR[c] (95 % CI[d])	*P*-value[e]
	CSF2 rs25881	***FLT1*** rs722503	***TLR9*** rs352140	***TFPI*** C-399T	**Controls**	**pPROM[b] cases**		
**APTT[f]**	T	T	-	-	0.118	0.187	5.51 (2.08 - 14.59)	**≤0.001**
	-	C	C	C	0.084	0.086	0.04 (0.00 - 0.66)	**0.026**
	-	T	C	C	0.280	0.302	3.44 (1.35 - 8.79)	**0.011**
	-	T	T	T	0.029	0.066	16.81 (2.07 - 136.29)	**0.009**
**PLT[g]**	T	T	-	-	0.118	0.187	1.65 (1.01 - 2.69)	**0.045**
	C	T	T	T	0.036	0.054	3.07 (1.01 - 9.31)	**0.048**
	-	T	T	T	0.029	0.066	3.04 (1.09 - 8.51)	**0.035**
**PCT[h]**	T	T	-	-	0.118	0.187	1.76 (1.07 - 2.88)	**0.026**
	-	T	T	T	0.029	0.066	3.06 (1.07 - 8.77)	**0.038**
**PLT+MPV[i]**	T	T	-	-	0.118	0.187	1.68 (1.02 - 2.75)	**0.041**
	-	T	T	T	0.029	0.066	3.02 (1.04 - 8.73)	**0.043**
	C	T	T	T	0.029	0.052	3.39 (1.01 - 11.31)	**0.048**
**PLT+PCT**	T	T	-	-	0.118	0.187	1.79 (1.08 - 2.95)	**0.024**
**MPV+PCT**	T	T	-	-	0.118	0.187	1.72 (1.05 - 2.84)	**0.034**
	T	T	T	-	0.079	0.134	2.07 (1.00 - 4.28)	**0.050**
	-	T	T	T	0.029	0.066	3.13 (1.07 - 9.13)	**0.038**
	C	T	T	T	0.029	0.052	3.63 (1.06 - 12.42)	**0.041**
	T	T	T	C	0.080	0.117	2.41 (1.05 - 5.51)	**0.038**
**PLT+MPV+PCT**	T	T	-	-	0.118	0.187	1.80 (1.09 - 2.98)	**0.022**
	C	C	-	C	0.180	0.233	1.65 (1.03 - 2.64)	**0.036**
	T	T	T	-	0.079	0.134	2.22 (1.06 - 4.63)	**0.035**
	-	T	T	T	0.029	0.066	3.21 (1.10 - 9.40)	**0.034**
	C	T	T	T	0.029	0.052	3.68 (1.07 - 12.61)	**0.039**
	T	T	T	C	0.080	0.117	2.64 (1.15 - 6.05)	**0.023**

**PLT+PDW[j]+MPV**	-	T	T	T	0.029	0.066	2.95 (1.02 - 8.52)	**0.046**
**PDW+MPV+PCT**	T	T	-	-	0.118	0.187	1.69 (1.02 - 2.80)	**0.041**
	-	T	T	T	0.029	0.066	3.08 (1.06 - 8.96)	**0.040**
	C	T	T	T	0.029	0.052	3.56 (1.03 - 12.23)	**0.045**
	T	T	T	C	0.080	0.117	2.34 (1.01 - 5.40)	**0.048**
**PLT+PDW+MPV+PCT**	T	T	-	-	0.118	0.187	1.76 (1.06 - 2.92)	**0.029**
	T	T	T	-	0.079	0.134	2.14 (1.02 - 4.52)	**0.046**
	-	T	T	T	0.029	0.066	3.20 (1.09 - 9.37)	**0.035**
	C	T	T	T	0.029	0.052	3.61 (1.04 - 12.52)	**0.044**
	T	T	T	C	0.080	0.117	2.55 (1.09 - 5.93)	**0.031**

[a.] SNP, single nucleotide polymorphism; [b.] pPROM, preterm prelabor rupture of membranes; [c.] OR, odds ratio; [d.] 95% CI, confidence interval; [e.] *P*-value, statistically significant results are marked in bold; [f.] APTT, activated partial thromboplastin time; [g.] PLT, platelet; [h.] PCT, plateletcrit; [i.] MPV, mean platelet volume; [j.] PDW, platelet distribution width.

Table S9. Differences in the prevalence of multiple-SNP variants for *CSF2*, *FLT1*, *TLR9*, and *TFPI* polymorphisms between women with term and preterm PROM, corrected for APTT and PLT parameters.

Categorical covariate	Polymorphisms / Alleles				Multiple-SNP[a] variant frequency		OR[d] (95 % CI[e])	*P*-value[f]
	CSF2 **rs25881**	***FLT1*** **rs722503**	***TLR9*** **rs352140**	***TFPI*** **C-399T**	**tPROM[b]**	**pPROM[c]**		
**APTT[g]**	-	C	T	C	0.041	0.148	4.54 (1.14 - 18.10)	**0.034**
	C	C	T	C	0.045	0.146	4.32 (1.07 - 17.48)	**0.042**
**PLT[h]**	-	C	T	-	0.061	0.152	3.56 (1.04 - 12.23)	**0.045**
	C	C	T	-	0.059	0.150	3.56 (1.03 - 12.33)	**0.047**
	-	C	T	C	0.041	0.148	3.92 (1.19 - 12.92)	**0.026**
**PDW[i]**	-	C	T	-	0.061	0.152	4.20 (1.16 - 15.26)	**0.030**
	C	C	T	-	0.059	0.150	4.62 (1.22 - 17.40)	**0.025**
	-	C	T	C	0.041	0.148	5.12 (1.42 - 18.39)	**0.013**
	C	C	T	C	0.045	0.146	5.28 (1.38 - 20.21)	**0.016**
**MPV[j]**	-	C	T	-	0.061	0.152	4.32 (1.19 - 15.70)	**0.028**
	C	C	T	-	0.059	0.150	4.79 (1.27 - 18.11)	**0.022**
	-	C	T	C	0.041	0.148	5.19 (1.44 - 18.66)	**0.013**
	C	C	T	C	0.045	0.146	5.39 (1.40 - 20.68)	**0.015**
**PCT[k]**	-	C	T	C	0.041	0.148	3.43 (1.02 - 11.51)	**0.047**
**PLT+PDW**	-	C	T	-	0.061	0.152	4.25 (1.17 - 15.43)	**0.029**
	C	C	T	-	0.059	0.150	4.62 (1.23 - 17.37)	**0.025**
	-	C	T	C	0.041	0.148	5.12 (1.43 - 18.38)	**0.013**
	C	C	T	C	0.045	0.146	5.28 (1.38 - 20.19)	**0.016**
	T	T	T	T	0.000	0.014	6.05 (4.71–7.78)	**≤ 0.001**
**PDW+MPV**	C	C	T	C	0.045	0.146	5.33 (1.39 - 20.48)	**0.016**
**PLT+PDW+MPV**	-	C	T	-	0.061	0.152	4.29 (1.18 - 15.57)	**0.028**
	C	C	T	-	0.059	0.150	4.68 (1.24 - 17.68)	**0.024**

	-	C	T	C	0.041	0.148	5.15 (1.43 - 18.50)	**0.013**
	C	C	T	C	0.045	0.146	5.34 (1.39 - 20.57)	**0.016**
**PLT+PDW+MPV+PCT**	-	C	-	C	0.149	0.234	2.14 (1.02 - 4.49)	**0.046**
	-	C	T	-	0.061	0.152	4.31 (1.21 - 15.31)	**0.025**
	C	C	-	C	0.147	0.233	2.26 (1.07 - 4.79)	**0.034**
	C	C	T	-	0.059	0.150	4.73 (1.28 - 17.45)	**0.021**
	-	C	T	C	0.041	0.148	5.63 (1.61 - 19.75)	**0.008**
	C	C	T	C	0.045	0.146	7.02 (1.84 - 26.81)	**0.005**

[a.] SNP, single nucleotide polymorphism; [b.] tPROM, term prelabor rupture of membranes; [c.] pPROM, preterm PROM; [d.] OR, odds ratio; [e.] 95% CI, confidence interval; [f.] *P*-value, statistically significant results are marked in bold; [g.] APTT, activated partial thromboplastin time; [h.] PLT, platelet; [i.] PDW, platelet distribution width; [j.] MPV, mean platelet volume; [k.] PCT, plateletcrit.

Table S10. Distribution of multiple-SNP variants for *CSF2*, *FLT1*, *TLR9*, and *TFPI* polymorphisms between women with term and preterm PROM, corrected for pregnancy disorders.

Pregnancy disorders	Polymorphisms / Alleles				Multiple-SNP[a] variant frequency		OR[d] (95 % CI[e])	*P*-value[f]
	***CSF2* rs25881**	***FLT1* rs722503**	***TLR9* rs352140**	***TFPI* C-399T**	**tPROM[b]**	**pPROM[c]**		
**Asthma and respiratory system infections**	-	T	T	C	0.394	0.369	1.00	---
	-	T	C	C	0.349	0.302	0.98 (0.50 - 1.93)	0.950
	-	C	T	C	0.041	0.148	3.46 (1.02 - 11.74)	**0.048**
	-	C	C	C	0.114	0.086	0.86 (0.32 - 2.36)	0.770
	-	T	T	T	0.072	0.066	1.25 (0.41 - 3.81)	0.690
	-	C	C	T	0.000	0.013	0.38 (0.05 - 3.15)	0.370
	C	T	T	C	0.288	0.255	1.00	---
	C	T	C	C	0.293	0.245	0.94 (0.43 - 2.07)	0.880
	C	C	T	C	0.045	0.146	3.32 (0.96 - 11.53)	0.060
	T	T	T	C	0.122	0.117	1.01 (0.42 - 2.42)	0.990
	C	C	C	C	0.106	0.088	0.94 (0.34 - 2.60)	0.910
	C	T	T	T	0.067	0.052	1.10 (0.33 - 3.68)	0.880
	T	T	C	C	0.045	0.054	1.49 (0.39 - 5.76)	0.560
	C	C	C	T	0.020	0.011	0.37 (0.05 - 2.63)	0.320
**Bleeding**	-	T	T	C	0.394	0.369	1.00	---
	-	T	C	C	0.349	0.302	0.98 (0.50 - 1.94)	0.960
	-	C	T	C	0.041	0.148	3.52 (1.04 - 11.85)	**0.044**
	-	C	C	C	0.114	0.086	0.86 (0.31 - 2.38)	0.780
	-	T	T	T	0.072	0.066	1.26 (0.41 - 3.86)	0.690
	-	C	C	T	0.000	0.013	0.35 (0.04 - 3.03)	0.340
	C	T	T	C	0.288	0.255	1.00	---

	C	T	C	C	0.293	0.245	0.95 (0.44 - 2.09)	0.910
	C	C	T	C	0.045	0.146	3.41 (0.99 - 11.74)	0.054
	T	T	T	C	0.122	0.117	1.03 (0.43 - 2.47)	0.950
	C	C	C	C	0.106	0.088	0.95 (0.34 - 2.63)	0.920
	C	T	T	T	0.067	0.052	1.11 (0.33 - 3.74)	0.860
	T	T	C	C	0.045	0.054	1.48 (0.38 - 5.74)	0.570
	C	C	C	T	0.020	0.011	0.34 (0.04 - 2.57)	0.300
**Diabetes mellitus**	C	T	T	-	0.359	0.299	1.00	---
	C	T	C	-	0.290	0.276	1.10 (0.51 - 2.38)	0.820
	T	T	T	-	0.119	0.134	1.39 (0.59 - 3.24)	0.450
	C	C	T	-	0.059	0.150	3.78 (1.05 - 13.64)	**0.044**
	C	C	C	-	0.126	0.089	0.78 (0.30 - 2.00)	0.600
	T	T	C	-	0.047	0.045	1.42 (0.39 - 5.21)	0.600
	-	T	T	C	0.394	0.369	1.00	---
	-	T	C	C	0.349	0.302	0.97 (0.50 - 1.91)	0.940
	-	C	T	C	0.041	0.148	3.71 (1.10 - 12.52)	**0.036**
	-	C	C	C	0.114	0.086	0.88 (0.32 - 2.45)	0.810
	-	T	T	T	0.072	0.066	1.19 (0.38 - 3.73)	0.760
	-	C	C	T	0.000	0.013	0.29 (0.03 - 2.82)	0.290
	C	T	T	C	0.288	0.255	1.00	---
	C	T	C	C	0.293	0.245	0.95 (0.44 - 2.09)	0.900
	T	T	T	C	0.122	0.117	1.10 (0.46 - 2.63)	0.840
	C	C	T	C	0.045	0.146	3.61 (1.04 - 12.52)	**0.045**
	C	C	C	C	0.106	0.088	0.98 (0.35 - 2.75)	0.970
	C	T	T	T	0.067	0.052	1.01 (0.29 - 3.50)	0.980
	T	T	C	C	0.045	0.054	1.54 (0.40 - 5.99)	0.530
	C	C	C	T	0.020	0.011	0.28 (0.03 - 2.50)	0.260
**Hypertension**	-	T	T	C	0.394	0.369	1.00	---
	-	T	C	C	0.349	0.302	0.99 (0.51 - 1.95)	0.980
	-	C	T	C	0.041	0.148	3.61 (1.06 - 12.25)	**0.041**

	-	C	C	C	0.114	0.086	0.85 (0.31 - 2.35)	0.750
	-	T	T	T	0.072	0.066	1.27 (0.41 - 3.90)	0.680
	-	C	C	T	0.000	0.013	0.35 (0.04 - 2.88)	0.330
	C	T	T	C	0.288	0.255	1.00	---
	C	T	C	C	0.293	0.245	0.96 (0.44 - 2.11)	0.920
	C	C	T	C	0.045	0.146	3.49 (1.00 - 12.14)	0.051
	T	T	T	C	0.122	0.117	1.03 (0.43 - 2.47)	0.940
	C	C	C	C	0.106	0.088	0.93 (0.34 - 2.60)	0.900
	C	T	T	T	0.067	0.052	1.12 (0.33 - 3.78)	0.850
	T	T	C	C	0.045	0.054	1.50 (0.39 - 5.79)	0.550
	C	C	C	T	0.020	0.011	0.34 (0.05 - 2.48)	0.290
**Hypothyroidism**	-	T	T	C	0.394	0.369	1.00	---
	-	T	C	C	0.349	0.302	0.99 (0.51 - 1.95)	0.980
	-	C	T	C	0.041	0.148	3.56 (1.06 - 11.97)	**0.042**
	-	C	C	C	0.114	0.086	0.87 (0.31 - 2.41)	0.790
	-	T	T	T	0.072	0.066	1.25 (0.41 - 3.81)	0.700
	-	C	C	T	0.000	0.013	0.37 (0.05 - 2.93)	0.340
	C	T	T	C	0.288	0.255	1.00	---
	C	T	C	C	0.293	0.245	0.95 (0.44 - 2.09)	0.910
	C	C	T	C	0.045	0.146	3.45 (1.00 - 11.89)	0.052
	T	T	T	C	0.122	0.117	1.02 (0.42 - 2.43)	0.970
	C	C	C	C	0.106	0.088	0.95 (0.34 - 2.65)	0.930
	C	T	T	T	0.067	0.052	1.10 (0.33 - 3.69)	0.880
	T	T	C	C	0.045	0.054	1.53 (0.40 - 5.92)	0.540
	C	C	C	T	0.020	0.011	0.35 (0.05 - 2.48)	0.290
**Serological conflict**	C	T	T	-	0.359	0.299	1.00	---
	C	T	C	-	0.290	0.276	1.11 (0.51 - 2.42)	0.780
	T	T	T	-	0.119	0.134	1.29 (0.56 - 2.96)	0.560
	C	C	T	-	0.059	0.150	3.78 (1.02 - 14.03)	**0.048**
	C	C	C	-	0.126	0.089	0.78 (0.30 - 2.01)	0.610

	T	T	C	-	0.047	0.045	1.34 (0.36 - 4.94)	0.660
	-	T	T	C	0.394	0.369	1.00	---
	-	T	C	C	0.349	0.302	1.01 (0.51 - 1.99)	0.980
	-	C	T	C	0.041	0.148	3.79 (1.09 - 13.25)	**0.038**
	-	C	C	C	0.114	0.086	0.89 (0.32 - 2.47)	0.820
	-	T	T	T	0.072	0.066	1.23 (0.40 - 3.76)	0.710
	-	C	C	T	0.000	0.013	0.36 (0.04 - 2.91)	0.340
	C	T	T	C	0.288	0.255	1.00	---
	C	T	C	C	0.293	0.245	0.99 (0.45 - 2.18)	0.980
	T	T	T	C	0.122	0.117	1.04 (0.44 - 2.48)	0.920
	C	C	T	C	0.045	0.146	3.71 (1.03 - 13.37)	**0.046**
	C	C	C	C	0.106	0.088	0.98 (0.35 - 2.74)	0.970
	C	T	T	T	0.067	0.052	1.09 (0.33 - 3.64)	0.890
	T	T	C	C	0.045	0.054	1.50 (0.39 - 5.73)	0.560
	C	C	C	T	0.020	0.011	0.34 (0.05 - 2.55)	0.300
**Threatened miscarriage**	C	T	T	-	0.359	0.299	1.00	---
	C	T	C	-	0.290	0.276	1.04 (0.48 - 2.27)	0.910
	T	T	T	-	0.119	0.134	1.29 (0.55 - 3.04)	0.560
	C	C	T	-	0.059	0.150	3.68 (1.04 - 13.06)	**0.046**
	C	C	C	-	0.126	0.089	0.69 (0.26 - 1.83)	0.460
	T	T	C	-	0.047	0.045	1.23 (0.34 - 4.51)	0.750
	-	T	T	C	0.394	0.369	1.00	---
	-	T	C	C	0.349	0.302	0.96 (0.49 - 1.88)	0.900
	-	C	T	C	0.041	0.148	3.87 (1.14 - 13.15)	**0.031**
	-	C	C	C	0.114	0.086	0.74 (0.26 - 2.10)	0.570
	-	T	T	T	0.072	0.066	1.25 (0.41 - 3.80)	0.700
	-	C	C	T	0.000	0.013	0.38 (0.05 - 2.95)	0.350
	C	T	T	C	0.288	0.255	1.00	---
	C	T	C	C	0.293	0.245	0.94 (0.43 - 2.06)	0.870
	C	C	T	C	0.045	0.146	3.67 (1.06 - 12.74)	**0.042**

T	T	T	C	0.122	0.117	1.08 (0.45 - 2.61)	0.870
C	C	C	C	0.106	0.088	0.85 (0.30 - 2.42)	0.760
C	T	T	T	0.067	0.052	1.15 (0.34 - 3.82)	0.820
T	T	C	C	0.045	0.054	1.42 (0.37 - 5.43)	0.610
C	C	C	T	0.019	0.011	0.36 (0.05 - 2.52)	0.300

[a.] SNP, single nucleotide polymorphism; [b.] tPROM, term prelabor rupture of membranes; [c.] pPROM, preterm PROM; [d.] OR, odds ratio; [e.] 95% CI, confidence interval; [f.] *P*-value, statistically significant results are marked in bold.
